# Assessing Mathematical School Readiness

**DOI:** 10.3389/fpsyg.2019.01173

**Published:** 2019-05-24

**Authors:** Sandrine Mejias, Claire Muller, Christine Schiltz

**Affiliations:** ^1^CNRS, CHU Lille, UMR 9193 – SCALab – Sciences Cognitives et Sciences Affectives, Université de Lille, Lille, France; ^2^Luxembourg Centre for Educational Testing , Université du Luxembourg, Esch-sur-Alzette, Luxembourg; ^3^Institute of Cognitive Science and Assessment, Université du Luxembourg, Esch-sur-Alzette, Luxembourg

**Keywords:** mathematical school readiness, numeracy, math skills, number sense, arithmetic, mathematical learning

## Abstract

Early math skills matter for later formal mathematical performances, academic and professional success. Accordingly, it is important to accurately assess mathematical school readiness (MSR) at the beginning of elementary school. This would help identifying children who are at risk of encountering difficulties in math and then stimulate their acquisition of mathematical skills as soon as possible. In the present study, we present a new test that allows professionals working with children (e.g., teachers, school psychologists, speech therapists, and school doctors) to assess children’s MSR when they enter formal schooling in a simple, rapid and efficient manner. 346 children were assessed at the beginning of 1st Grade (6-to-7-year-olds) with a collective test assessing early mathematical abilities (T1). In addition, children’s math skills were evaluated with classical curriculum math tests at T1 and a year later, in 2nd Grade (T2, 7-to-8-year-olds). After assessing internal consistency, three tasks were retained for the final version of the MSR test. Test performance confirmed to be essentially unidimensional and systematically related to the scores children obtained in classical tests in 1st and 2nd Grade. By using the present MSR test, it is possible to identify pupils at risk of developing low math skills right from the start of formal schooling in 1st Grade. Such a tool is needed, as children’s level in math at school beginning (or school readiness) is known to be foundational for their future academic and professional carrier.

## Introduction

Considering the importance of mathematics in modern society, math activities play a central role in a child’s education. Building good math skills is an essential part of a first grader’s learning process and determines academic success down the road. Indeed, it has been demonstrated that children’s scholastic level at the beginning of formal schooling - or school readiness - is very important for their future academic and professional carriers (Currie and Thomas, 1999; Duncan et al., 2007; Romano et al., 2010). Especially, early math skills developed during kindergarten appear to be one of the most powerful predictors of later formal learning, including reading (Duncan et al., 2007; Pagani et al., 2010; Romano et al., 2010). In addition, many longitudinal studies have emphasized the importance of early math skills for the development of more elaborate mathematical abilities (e.g., Jordan et al., 2007, 2009; Aunio and Niemivirta, 2010). Moreover, young adults’ proficiency to use simple math to solve problems encountered in everyday life seems to determine their likelihood of full-time employment (e.g., Rivera-Batiz, 1992).

Consequently, it seems highly relevant to evaluate early math skills of children at the beginning of formal (mathematical) learning, or in other words, their mathematical school readiness (MSR). This allows us to identify children with low math skills at the beginning of 1st Grade and to subsequently set up appropriate educational support measures for the children in need (National Research Council, 2001). These support measures will provide an ideal basis for later mathematical learning and prevent a vicious circle of poor basic skills leading to poor mathematical learning, which in turn results in numerical shortcomings. However, to be able to identify children in (great) difficulty, teachers need validated and standardized tools. Yet, they deplore the lack of such tools and indicate that they are usually forced to rely on their own “home-made” tools or intuition, which is not ideal and leaves them feeling uncomfortable (Do, 2007). Data showing that teachers’ judgments are perceptually biased and that they have difficulties judging their students’ cognitive potential confirm the actual problem of the situation (e.g., Fischbach et al., 2013).

Mathematical school readiness focuses on the narrow window of math development between the acquisition of (pre)mathematical precursor skills in kindergarten and the implementation of formal mathematical education in elementary school. The acquisition of the Arabic number notation system constitutes a key element of MSR, because it bridges the innate core magnitude system (e.g., Feigenson et al., 2004) and the development of the exact number representations underlying the (ordinal) mental number line and arithmetic thinking (see von Aster, 2000; von Aster and Shalev, 2007). According to von Aster and Shalev’s (2007) four-step-developmental model of number acquisition, the acquisition of the Arabic number system (i.e., the visual Arabic code, see also Dehaene, 1992) is a major challenge in the development of children’s math skills. This acquisition implies the progressive learning of visual number symbols (i.e., Arabic numbers), the place value syntax and the corresponding transcoding rules (see Thevenot and Fayol, 2018, for a review). Together with the verbal number system, which develops during preschool years, the acquisition of the Arabic notation system (including multi-digit numbers) implicitly starts in preschool (Gilmore et al., 2007; Mejias and Schiltz, 2013), probably because of the widespread use of digital displays in children’s direct environment. It is then systematically consolidated and enhanced during 1st Grade through formal and explicit instruction (Mix et al., 2014).

However, poorly developed mathematical competencies are observed in a non-negligible number of (young) children and adults (3–7%; GrossTsur et al., 1996; Shalev et al., 2005; Reigosa-Crespo et al., 2012; American Psychiatric Association [APA], 2013). According to the DSM-5 (2013), specific learning disorder is now a single, overall diagnosis, incorporating deficits that impact academic achievement. Specific learning disorder refers to significant and persistent difficulties in learning and using one’s cultural symbol systems (e.g., alphabet, characters, and Arabic numbers) that are required for skilled reading, writing, and math, and must be learned by instruction. Persons with specific learning disorder are unable to perform academically at a level appropriate to their intelligence and age. The definition states that difficulties should have persisted for at least 6 months despite interventions, and skills should be substantially below those expected for that given age. It is now recommended to give this diagnosis only from 1.5 standard deviations below the mean for age (which correspond to a performance within the lowest 7% in standardized mathematical tests, while previously the lowest 10% was commonly accepted). Beside those children with *specific learning disorder in math*, individuals achieving between 11% and 25% in standardized mathematical tests are classically identified as *low math achievers* (see Geary, 2011, for a review).

Low math achievers and especially individuals with specific learning disorder in math already perform less accurately than typically developing children in 1st Grade (Geary, 2011). Moreover, those children who perform in the lowest quartile in curriculum math tests also experience difficulties in basic math skills. This includes the processing of numbers and numerosities (Koontz and Berch, 1996; Landerl et al., 2004; Rubinsten and Henik, 2005; Mussolin et al., 2010), even when the task simply requires to count small sets of 1 to 4 items (Willburger et al., 2008). Furthermore, there are small but systematic group differences between 1st Grade children with specific learning disorder in math and controls in number naming, number writing (Geary et al., 1999) and comparing the magnitude of one- or two-digit numbers (Landerl et al., 2004, 2009; Rousselle and Noël, 2007; Iuculano et al., 2008; Landerl and Kölle, 2009). In sum, low math achievers and children with specific learning disorder in math reveal atypical performances in tasks requiring identification, representation and production of numerical quantities and symbols, in number comparison, in counting as well as in (simple) math problems.

There are currently a number of curriculum math tests for young children, which aim to provide an exhaustive diagnoses of mathematical learning disabilities (e.g., Van Nieuwenhoven et al., 2008; Lafay and Helloin, 2016) or to rapidly screen children’s ability in magnitude comparison (Nosworthy et al., 2013; Brankaer et al., 2017), which is one of the major precursors of math skills (Halberda et al., 2008). The former offer very complete and detailed insights into a child’s mathematical ability, but they have to be administered by specifically qualified professionals (i.e., psychologists or speech therapists) in time-consuming individual testing sessions. The latter can be easily and quickly run in group settings by a wide range of school professionals, but they focus on a specific mathematical precursor ability and also require a specific psychological knowledge basis for interpreting the results and translating them into classroom practice. In contrast, there are currently no tests that allow school teachers to evaluate children’s early mathematical abilities, by administrating and interpreting a validated and standardized test in the classroom setting. This is especially relevant and desirable at the beginning of formal schooling, because it allows teachers to identify those children with insufficient math skills directly at the beginning of the formal learning trajectory. Accordingly, teachers will be able to (a) set up appropriate learning and catch-up measures and/or (b) orient children toward special care. In summary, to the best of our knowledge, there are currently no tests that allow teachers to assess MSR based on psychometrically validated tasks with a high face-validity that can be easily administered in classroom settings.

Here we propose a test of MSR systematically assessing the mastery of visual number symbols at the entrance of formal schooling (i.e., at the beginning of 1st Grade). By this means, we intend to provide a psychometrically validated tool that can be easily used in classroom-settings and interpreted by school teachers. The MSR test therefore consists of different tasks having a high face-validity in the context of math education, while being also firmly embedded in neuro-psychological theories of typical and atypical numerical development. The test is composed of tasks probing Arabic number identification, writing Arabic numbers to dictation, writing Arabic numbers as a result of counting, Arabic number comparison, as well as basic arithmetic problem solving.

The present study aimed to evaluate the psychometric validity of the MSR test and its constituent tasks. Moreover, it determined the concurrent and predictive criterion validity of the test by evaluating whether 1st Graders’ performances on the test could significantly predict their performances on formal mathematics tasks, evaluated at the time of testing and 1 year later in 2nd Grade of elementary school. If the test items are valid and allow predicting children’s mathematical performance in 2nd Grade, then our test can help school teachers identify those children with insufficient MSR, thereby providing them with an empirical basis to orient these children toward dedicated educational support and special care measures.

## Materials and Methods

### Participants

Totally 346 participants (163 boys) were included in the study. The mean age was 6.30 years [± 0.35].

Participants were recruited from twelve different public schools in Belgium, at the beginning of 1st Grade.

This study was carried out in accordance with the recommendations of the research ethics committee of the Université Catholique de Louvain (Belgium). The protocol was approved by the research ethics committee of the Université Catholique de Louvain (Belgium). Written informed parental consent was obtained for each of the children, in accordance with the Declaration of Helsinki.

The schools’ socio-economic index level ranged from 4 to 20^1^. Participating schools were distributed in five different socio-economic index levels: One school was classified at very low level “4” (including 12 participants); two schools at intermediate level “12” (including 61 participants); four schools at intermediate level “13” (including 93 participants); four schools at very high level “19” (including 166 participants), and one school at highest level “20” (including 14 participants). In each school, children were tested for the first time (T1) in mid-September (6-to-7-year-olds) at the beginning of 1st Grade and for the second time (T2) in mid-September 1 year later (7-to-8-year-olds) at the beginning of 2nd Grade. Children’s age in months was similar across the five different socio-economic groups (with the largest age-difference in terms of months between two groups belonging to socio-economic index 12 and 13, *p* = 0.08; all other contrasts, *p* > 0.3). Data were collected by only one person.

Children who took part in the study had no history of developmental disorders and were considered as typically developing children by the Belgian psycho-medico- social services.

### Materials and Procedure

#### Mathematical School Readiness Test

To assess children’s MSR when they enter formal schooling a collective test of early mathematical abilities was developed. Considering the neuro-cognitive literature on typical and atypical numerical development (e.g., von Aster and Shalev, 2007; Dowker, 2008; Geary, 2011) this test aims to describe children’s abilities focusing on the mastery of visual number symbols typically required at the moment of formal (math) schooling entrance (i.e., at T1, during the first month of the 1st Grade). The test was designed to have a high face-validity for teachers and therefore includes all early math abilities described also in the school competence standards in Wallonia in Belgium (Van Lint, 2010) (i.e., visual number symbol identification, writing numbers to dictation, symbolic quantity representation, counting abilities and arithmetic abilities). The time required to complete the entire test was approximately 20 min. Five tasks were administered and evaluated (Appendix A):

(1) *Identifying visual number symbols (number identification)*: Arabic digits (3, 6, 8, and 9) were presented on a sheet of paper amongst non-numerical stimuli (a, @, $, and f). Children were asked to circle Arabic numbers (*N* = 4) and to cross out if not a number (*N* = 4). Note that if the child clearly identifies the numbers by circling them and does not cross out the other symbols, a maximum score of 8 is given. For example, a child who circles all the numbers and another non-numerical symbol is given a score of 7, etc. Average time required to achieve the subtest was 90 s.
(2) *Writing numbers to dictation (number writing)*: Children were told to write down the number they hear in the correct box (boxes were presented sequentially in line, with each box having a different color to prevent children from getting lost (e.g., “write 4 in the blue box (…), now write 7 in the orange box, …”). Six single-digit (4, 7, 1, 6, 5, and 9) and 4 two-digit numbers (10, 11, 13, and 16) were orally presented. Maximum score is 10 and the point is awarded even if the number is mirrored. Average time required to achieve the subtest was 180 s.
(3) *Comparing visual number symbols (number comparison)*: Children were asked to circle the largest of the two presented Arabic digits. A total of 12 pairs of numbers ranging from “2” to “420” were presented (Four pairs of one-digit numbers with a difference of 1 to 6; Six pairs of two-digit numbers with a difference of 1 to 50; Two pairs of three-digit numbers with a difference of 17 and 69; and see section “Appendix A” for the detailed version of the task). An example was given on the classroom’s blackboard with “1” and “2.” The maximum score that can be obtained in this task is 12. Average time required to achieve the subtest was 210 s.
(4) *Writing number symbols resulting from the counting of visual collections (counting)*: Children were asked to count four collections of 5 to 9 elements presented in an orderly manner (e.g., bunnies presented in a line) or presented in a disorderly manner (e.g., turtles presented in a scattered manner) and write down their answers. In the latter task three different collections were presented, two comprising the same sort of animal (turtles and sharks, respectively) and one with mixed animal sorts (lions and turtles). The maximum score that can be obtained in this task is 4 and the point is awarded even if the number is mirrored. Average time required to achieve the subtest was 120 s.
(5) *Solving basic arithmetic problems (arithmetic problem solving)*: Children were asked to resolve a maximum of *simple additions* presented as “houses” of 4, 5, and 6 (*N* = 18). Operators and results of the arithmetic problems ranged between (0 and 6). The maximum score that can be obtained in this task is 18 and the point is awarded even if the number is mirrored. Average time required to achieve the subtest was 6 min.
  Correct answers were scored as 1, wrong answers as 0.

#### Classical Mathematical Tests

To assess children’ formal mathematical skills when entering primary schooling (i.e., at T1, simultaneously with the MSR test administration) and after one entire year of formal schooling (i.e., at T2, during the first month of 2nd Grade), children were given two different classical mathematical screening tests.

##### The arithmetic number fact test

Tempo Test Rekenen, TTR; De Vos (1992) this test consists in two lists of arithmetic number fact problems, consisting of additions and subtractions, respectively. Children have to solve as many operations as possible within 1 min per condition. There are enough operations planned so that the child does not reach the end of the test in 1 min. Correct answers were scored as 1, wrong answers as 0. A child’s total score in the *Arithmetic Number Fact Test* corresponded to the sum of the scores obtained in the respective tasks. The TTR test was administered at T1 and T2.

##### The kortrijk arithmetic test

Kortrijkse Rekentest-Revisie, KRT-R; Baudonck et al. (2006) this standardized test measures children’s mathematical abilities through two subscales, these subscales correspond to the mental arithmetic computation (e.g., 43 + 36 = …) and the number system knowledge (e.g., 99 comes just after …) and are both scored on a maximum of 30 points. Correct answers were scored as 1, wrong answers as 0. There is no time limit to accomplish the test. The maximum score that can be obtained on this scale is 60. The KRT test was administered at T2 only as children need several months of formal schooling before this test can be administered.

## Statistical Analysis

The entire sample size could not be included in the following consistency analyses due to partial loss of data describing participants’ performance in each item. Accordingly, they were based on 158 out of 346 participants. The entire sample size is included in the other analyses. Collected data is avalaible in the Supplementary Material.

### Reliability

Reliability was measured by assessing internal consistency for each of the five tasks (number identification, number writing, number comparison, counting, and arithmetic problem solving) through Cronbach’s alpha and corrected item-total correlations. The corrected item-total correlation is the correlation of a selected item in one dimension with the other remaining items of that dimension. The impact of items on internal consistency was assessed by using Cronbach’s alpha with one-at-a-time deletion procedure. Cronbach’s alpha is expected to exceed 0.7 (Nunnally and Bernstein, 1978). We will consider this criterion as satisfied if 95% confidence intervals touch 0.7. Should a task not withstand the criterion, it will be excluded from further analyses.

### Validity

We evaluated construct, convergent and criterion validity. Construct validity refers to the degree to which a test measures what it claims to be measuring. Convergent validity is the degree to which measures of constructs that should theoretically be related, are in fact related. Criterion validity is the extent to which a test result can be used to predict the outcome of interest.

#### Construct Validity

We assume that the competence underlying performance on the MSR test is essentially unidimensional and can thus be summarized in one total score. We evaluated this assumption by looking at the interrelation of all psychometrically valid MSR tasks. We expected the Pearson correlation coefficients to be positive and significant. Unidimensionality was further assessed with principal component analysis (PCA). We expected that, adhering to the Kaiser (1960) criterion (keep only components with an Eigenvalue above 1), only 1 factor would be retained.

#### Convergent Validity

An overall performance score for the MSR test was computed as the average of the POMP (percent of maximum performance) of all psychometrically valid tasks. In order to assess convergent validity, we computed Pearson correlations of this score with the classical mathematical tests (CMAS), composed of TTR proposed at T1 and T2, and the KRT proposed at T2.

#### Criterion Validity

As individuals achieving below the 25th percentile in standardized mathematical tests are classically identified as *low math achievers* (Geary, 2011), we can suppose that children in this lower quartile are at risk of developing low mathematical abilities. Moreover, children achieving at the lowest 7% should be at risk of *specific learning disorder in math*.

We created one combined indicator for students’ mathematical ability at T2 (combined mathematical ability score; CMAS): TTR at T2 and KRT scores were both standardized and a sum score was created of these 2. Based on this sum score and the thresholds mentioned above, students were classified as “not at risk” (performance above the 25th percentile), “low math achievers” (7th percentile – equal to or below 25th percentile), or “potential specific learning disorder in math” (equal to or below 7th percentile).

For these groups we compared mean MSR scores using a one-way ANOVA and a *post hoc* Tukey test, expecting to find that scores would tend to be gradually lower from students “not at risk” over “low math achievers” to “potential specific learning disorder in math.” Additionally, using cross tables, we compared the probability of identifying students classified as “potential specific learning disorder in math” during T2 with our MSR test to the probability of identifying them with the TTR at T1, using the 25th percentile criterion.

Using multiple linear regression, we finally checked whether students’ performance on the MSR test explained variance in their score on the KRT at T2 over and above that explained by their performance on the TTR at T1.

Statistical analyses were performed using RStudio version 1.0.136.

## Results

Item difficulty ranges, mean POMP scores per task as well as the POMP score ranges can be found in Table 1.

**TABLE 1:** Task performance.

**Task**	**N**	**M POMP (SD)**	**POMP Range**		**Item difficulty range**	**Dropped**	**Internal consistency**	
			**Theor.**	**Emp.**	**%correct**		**α (95% CI)**	**r cor.**
Number identification	158	0.98 (0.06)	0–1	0.63–1	96 – 100	3, 6, 8	0.53 (0.43 – 0.64)	−0.031 –.0.59
Number writing	158	0.87 (0.13)	0–1	0.4–1	38 – 100	1, 4, 5	0.63 (0.55 – 0.75)	0.35 – 0.60
Number comparison	158	0.74 (0.19)	0–1	0.08–1	36 – 96	/	0.70 (0.64 – 0.77)	0.25 – 0.68
Counting	158	0.95 (0.12)	0–1	0.5–1	91 – 97	/	0.11 (−0.11 –0.34)	−0.07 – 0.53
Arithmetic problem solving	158	0.66 (35)	0–1	0–1	50 – 82	/	0.95 (0.94 – 0.96)	0.56 – 0.82

*POMP, Percentage of maximum performance; CI, confidence interval; Dropped, items that were dropped from internal consistency analysis due to lack of variance (all answered correctly).*

### Reliability

The Cronbach’s alpha coefficients for the five tasks are reported in Table 1 and range from 0.11 to 0.95. Due to low internal consistencies of the tasks “number identification ” and “counting,” these two tasks were dropped from further analyses. For the three remaining tasks, corrected item-total correlations coefficients were all *r*(156) ≥ 0.25, *p* < 0.01.

### Validity

#### Construct Validity

**FIGURE 1:**
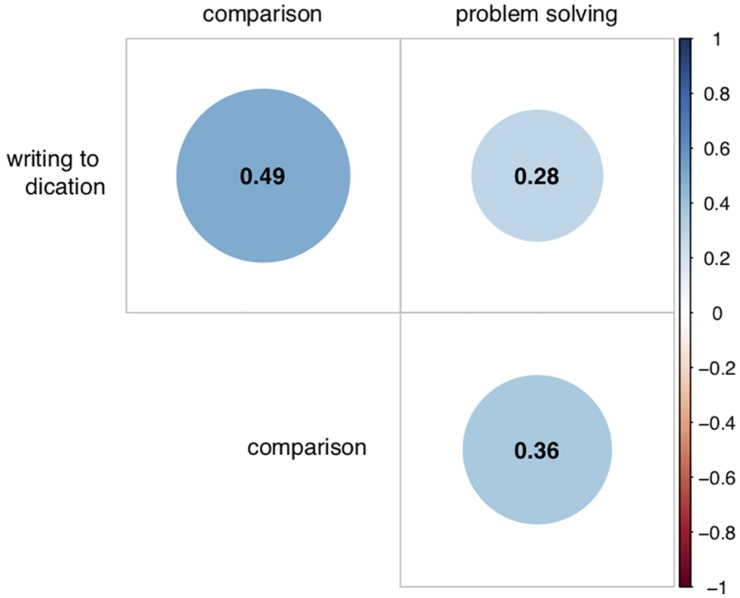
Correlation plot for relationship between the three valid MSR tasks. Coefficients represent Pearson correlations. All significant at *p* < 0.001.

Pearson correlations between the three remaining tasks were all positive and highly significant (see Figure 1). Effect sizes ranged from medium *r*(344) = 0.28 to large *r*(344) = 0.49, with *p* < 0.001 for each correlation coefficient.

The Kaiser-Meyer-Olkin measure of sampling adequacy was 0.62, so above the commonly recommended value of 0.6, and Bartlett’s test of sphericity was significant (χ2 (3) = 147.34, *p* < 0.001). Communalities were all well above 0.3. Given these indicators, PCA was deemed to be suitable. Eigenvalues of the extracted components were 1.76, 0.74, and 0.50, with the first factor explaining 59% of the total variance. As expected, only one factor is to be retained according to the Kaiser criterion.

#### Convergent Validity

**FIGURE 2:**
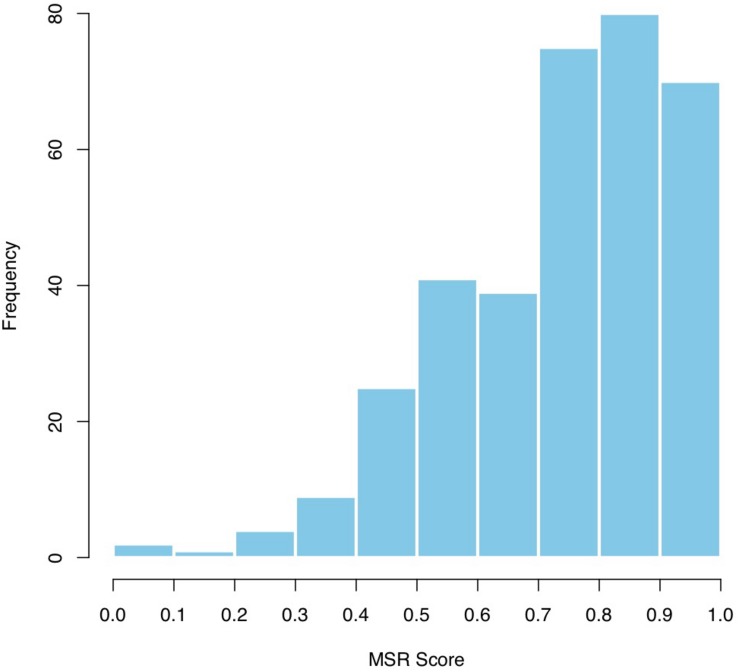
Distribution of MSR score in the sample of the study (*n* = 346).

Since PCA confirmed that one factor explains the majority of the variance in task performance for number writing, number comparison and arithmetic problem solving, an overall score in the MSR test was computed as the average of the POMP of the three tasks. The mean score of the test (*n* = 346) was *M* = 0.74, *SD* = 0.26, with a minimum equal to 0.03 and a maximum of 1. Distribution of the score is depicted in Figure 2.

**FIGURE 3:**
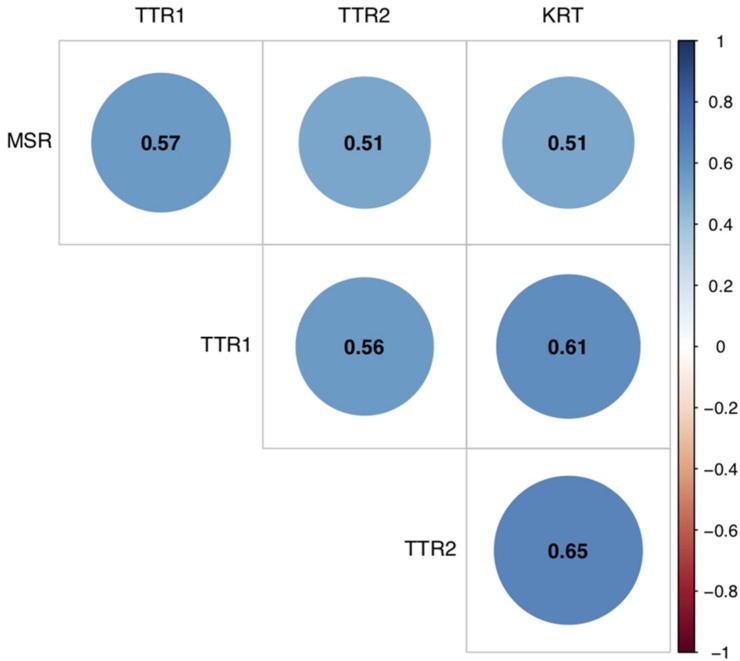
Correlation plot for relationship between MSR score and classical mathematical tests (CMAS) (TTR at T1 and T2, and KRT). Coefficients represent Pearson correlations. All significant at *p* < 0.001.

The MSR score significantly correlated with TTR at T1 *r*(344) = 0.57, *p* < 0.001 TTR at T2 *r*(344) = 0.51, *p* < 0.001 and KRT at T2 *r*(344) = 0.51, *p* < 0.001, see Figure 3.

#### Criterion Validity

**FIGURE 4:**
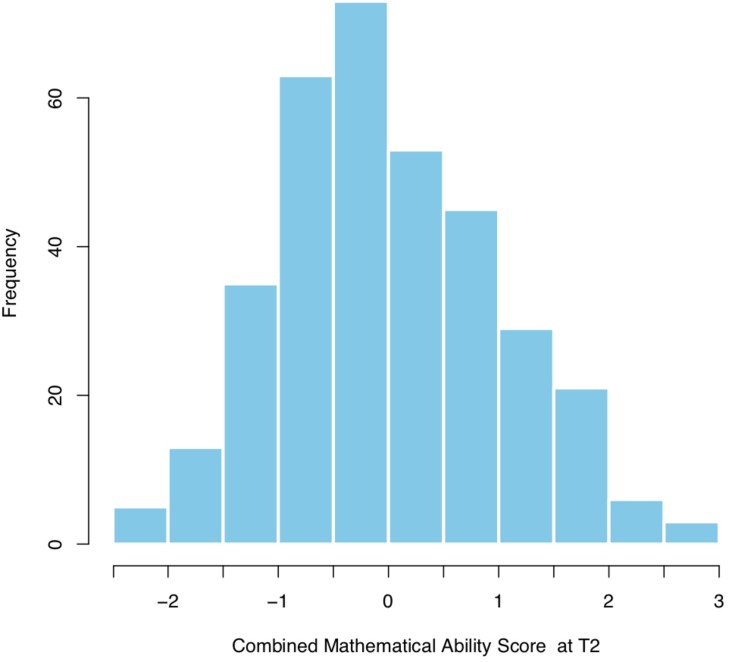
Distribution of Combined mathematical ability score (CMAS) at T2 in the sample of the study (*n* = 346).

**FIGURE 5:**
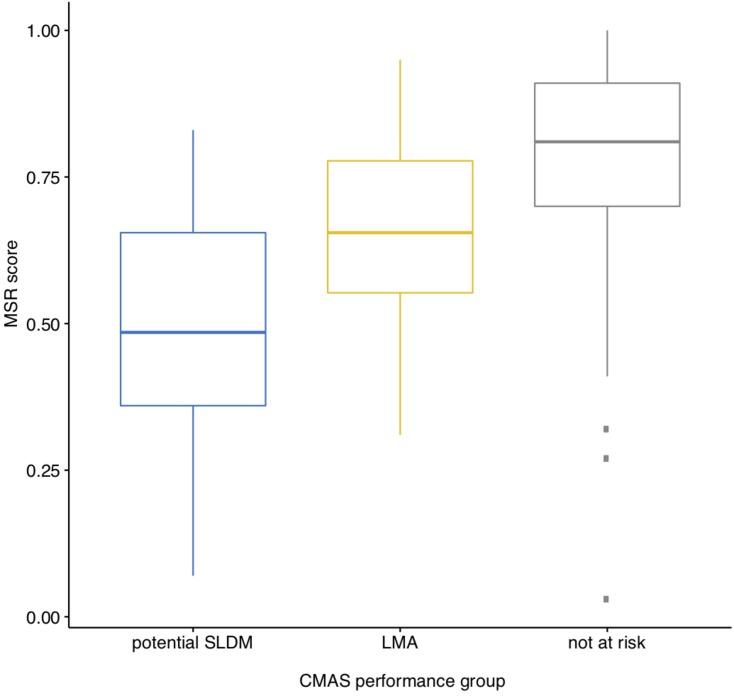
Boxplots for MSR score by CMAS performance group.

The distribution of the CMAS (centered and standardized) is presented in Figure 4. The Pearson correlation between CMAS and MSR is *r*(344) = 0.56, *p* < 0.001. The boxplots in Figure 5 visualize the finding that, as expected, MSR scores tend to be lower for students classified as “potential specific learning disorder in math.” For the students classified as “low math achievers,” scores tend to be somewhat better, but still lower than for students that were identified as “not at risk.” A one-way between subjects ANOVA confirms that mean scores of the 3 performance groups are significantly different with a large effect size *F*(2, 343) = 50.94, *p* < 0.001, η_p_^2^ = 0.23. A *post hoc* Tukey test showed that, as expected, “low math achievers” (*M* = 0.65, *SD* = 0.15) performed significantly better (*p* < 0.001) than students with a “potential learning disorder in math” (*M* = 0.50, *SD* = 0.19), but significantly worse (*p* < 0.001) than students “not at risk” (*M* = 0.79, *SD* = 0.16).

Table 2 presents the cross tabulation for performance grouping at T1 (with NRS and TTR) and T2 (CMAS). Using the MSR, 69% of students (compared to 66% with the TTR) classified as “potential specific learning disorder in math” at T2 were identified at least as “low math achievers” at T1 and 42% (compared to 31%) were already identified as “potential specific learning disorder in math.” Only 3% of students identified as “potential specific learning disorder in math” with the MSR were later classified as “not at risk” (compared to 7% with the TTR).

**TABLE 2:** Cross table for CMAS performance groups with MSR and TTR T1 performance groups.

		**MSR and TTR T1 performance groups**						
		**Potential specific learning**		**low math**		**Not at risk**		
		**disorder in math**		**achievers**				
		**MSR**	**TTR1**	**MSR**	**TTR T1**	**MSR**	**TTR T1**	**Row total**
CMAS performance groups	Potential specific learning disorder in math	11 (42)	8 (31)	7 (27)	9 (35)	8 (31)	9 (35)	26
	low math achievers	9 (15)	10 (16)	19 (31)	19 (31)	34 (55)	33 (53)	62
	Not at risk	8 (3)	17 (7)	35 (14)	27 (11)	215 (83)	214 (83)	258
	Column total	28	35	61	55	257	256	346

*Values represent counts with row percentages in brackets.*

Multiple regression was used in order to determine whether the MSR score would explain variance within CMAS performance over and above the variance explained by the TTR score at T1 (CMAS ∼TTR T1 + MSR). Results indicate that together MSR and TTR at T1 explain 41% of the variance within CMAS at T2 (*R*^2^ = 0.47, *F*(2, 343) = 151.5, *p* < 0.001) with highly significant contributions from both indicators (β_TTR_ = 0.47, *p* < 0.001 and β_NSR_ = 0.30, *p* < 0.001). Performance on the MSR test thus explains additional variance in mathematical ability on T2.

## Discussion

Children’s academic level at school entrance, i.e., their school readiness, is very important for their future academic success and professional career (Duncan et al., 2007). Detailed knowledge about children’s early abilities allows optimal adaptation of learning and instruction to their individual needs. It is therefore critical to accurately and efficiently assess school starter’s abilities in the core domains of schooling, such as mathematics.

The present study aimed to design a test that allows teachers or any professional working with children (e.g., school psychologists, speech therapists, school doctors) to assess young children’s MSR when they enter formal schooling in a simple, rapid and efficient manner. Such a MSR test should provide insights into children’s numerical abilities at the beginning of the 1st Grade by revealing their strengths and/or weaknesses, thereby allowing for the anticipation of their later achievements and/or problems in mathematics. The test aims to differentiate between children with distinct math ability levels, focusing in particular on the identification of children with performances in the lower range. Importantly, it is not a neuro-psychological test battery allowing full-fledged diagnosis but the test aims to inform teachers and interested professionals about children’s early mathematics skills to guide their future educational set-up and/or orientation toward specific diagnosis and care measures on a solid evidence basis.

The tasks included in the test systematically related to theories of neuro-cognitive development as well as to academic competence standards, thereby ensuring that children’s early mathematical abilities are measured in a cognitively accurate and valid manner. In addition, they are easy to use and can be readily interpreted by teachers. The initial test version included 5 tasks assessing children’s mastery of visual number symbols: identifying visual number symbols, writing numbers to dictation, comparing visual number symbols, writing number symbols resulting from the counting of visual collections, as well as solving basic arithmetic problems. After carefully assessing the internal consistency of the different tasks, the final and validated test version retained three tasks: writing numbers to dictation, comparing visual number symbols and solving basic arithmetic problems. Internal consistency indeed indicated that the tasks consisting in identifying visual number symbols and in writing number symbols following the counting of visual collections needed to be excluded. Those two tasks lacked sensitivity and demonstrated a very low internal consistency. At the first stage of test construction, it seemed important to include the number identification task as it corresponds to a basic entry-level skill, preceding the ability to read and understand visual symbolic numbers. The same applies to the counting task, in which visual collections that had to be counted consisted of 5 to 9 elements. These type of tasks have been used in well-known diagnostic tests such as the TEDI-Math (Van Nieuwenhoven et al., 2008). The TEDI-Math is used for diagnosis of numerical learning disorders from the end of the 2nd year of kindergarten to the end of 3rd Grade of elementary school. Yet, the fact that the number identification task was not a sensitive measure at the beginning of 1st Grade is not surprising considering that children in kindergarten (from 4-to-6-year-olds) revealed remarkably good knowledge of visual number symbols. They are thus able to estimate, compare, add and subtract 2-digit numbers, based on their approximate number sense (Gilmore et al., 2007; Mejias and Schiltz, 2013). Concerning the present counting task, it appeared to be much simpler than the task proposed in the TEDI-Math, in which the child must provide an answer based on a detailed understanding of elaborate language instructions.

Considering the final version of the MSR task comprising the three tasks “number writing,” “number comparison,” and “arithmetic problem solving” a PCA indicated that the final test can be characterized by a single dimension involving basic number skills. The three internally consistent tasks of the MSR test (as all corrected item-total correlations were greater than 0.25) were not redundant, indicating that all subtests contribute to the measure of early mathematical abilities. Performance on all three tasks thus contributed relevant information to explaining individual differences in early mathematical abilities, which are considered to be essential scaffolds for later formal arithmetical abilities. It was indeed proposed that mathematical abilities develop quasi-hierarchically, with more mature and complex mathematical knowledge building up on more basic skills (Claessens et al., 2009; Jordan et al., 2009; Claesens and Engel, 2013; Watts et al., 2014; Aunio and Räsänen, 2016). The test thus notifies about the mastery of visual symbols, by providing information about children’s abilities to write numbers to dictation (i.e., referring to transcoding abilities; e.g., Molfese et al., 2006), to compare Arabic digits (i.e., referring to number magnitude representations and place-value understanding; e.g., Nosworthy et al., 2013; Brankaer et al., 2017) and to solve basic arithmetic problems (i.e., referring to basic computational skills; e.g., Jordan et al., 2006).

The scores in the MSR test were distributed over the entire performance range, going from very low (0.03) to perfect (1.0). This indicates that the difficulty level of the test is well adapted to capture the performance of all children attending 1st Grade. Critically, children’s performances on the MSR test at school entrance predicted their mathematical performances 1 year later, yielding a correlation of 0.56 with a combined measure of two CMAS. Moreover, the 42% of children, who were identified as “potential specific learning disorder in math” since they scored below 7% in the MSR test (i.e., 3% of the total group), also achieved below 7% 1 year later in 2nd Grade. In comparison, only 31% of the children were accurately classified based on the TTR1. The present test therefore allows to anticipate later mathematics achievements, which in turn facilitates early actions specifically adapted to a child’s profile. Especially, those children scoring below 7% run the risk of significantly falling behind if no specifically dedicated measures are taken. They should therefore be oriented toward further psychological support and special needs education, if the specific learning disorder in math is confirmed by classical neuro-psychological test procedures. Nevertheless, a certain number of classification errors can arise with the novel MSR test, as with the more established tests TTR and KRT. These might reflect for instance math problems arising after the first assessment point T1, or measurement noise occurring at T1 or T2 and which leads to performances that do not truly reflect children mathematical abilities (i.e., tiredness, lack of concentration or lack of motivation during test taking).

In line with previous studies in preschoolers (Duncan et al., 2007; Aunio and Niemivirta, 2010; Pagani et al., 2010; Romano et al., 2010) the present results confirm that early numeracy performances are a good predictor of later more elaborate math performance. Mastery of the Arabic number system is a major challenge in math skill acquisition, as it emerges from the progressive association of numerical meaning to visual symbols, which takes place over a 2–3 year period from age 3 onward (see Thevenot and Fayol, 2018, for a review). This corresponds to the 3rd and 4th stage of the von Aster and Shalev (2007); see also Kaufmann et al., 2014), referring to the mastery of Arabic number representation and their ordering on a mental number line, respectively. From a formal point of view, these acquisitions classically emerge through explicit academic learning during kindergarten and are subsequently reinforced in primary school. Since approximately 30 years (with the widespread use of digital displays), implicit learning of visual number symbols also often occurs in children’s home environment (Thevenot and Fayol, 2018). The more children are exposed to informal learning opportunities at home, the better they perform on basic number skill tasks (Melhuish et al., 2008; Benavides-Varela et al., 2016). Accordingly, some of the children scoring below 25% may be those lacking informal number activities, therefore lagging behind peers, who experience more informal numerical activities in their early home environment (see Ramani and Siegler, 2014, for a review). Activities that include Arabic numbers have been shown to help these children overcome their gap (e.g., Ramani and Siegler, 2008; Siegler and Ramani, 2008). Apart from lacking numerical stimulation, some children scoring below 7% may additionally suffer from specific learning disorder in math, therefore requiring even more targeted follow-ups. The MSR does not allow disentangling these two problem sources. Yet in either case, it is important to be able to efficiently and reliably identify children as soon as possible.

As opposed to existing tools (i.e., exhaustive test batteries, minimal screeners), our MSR test aims to assess children’s early mathematical abilities, while being easy to administer as well as readily interpretable by any early childhood professional such as teachers, school psychologists, speech therapists, and school doctors. The present test version is, however, limited to (pre)-school curricula including explicit instruction of number symbols up to 10 and using French as instruction language. In future studies norms should be collected in French-speaking countries with similar curricula. Furthermore, it will be important to address potential performance influences due to children’s socio-economic and (multi-)lingual environment (e.g., Mejias and Schiltz, 2013; Van Rinsveld et al., 2015).

In sum, the MSR test offers a tool that is short (approximately 15 min), can be administered individually or collectively in the classroom setting and allows to reliably evaluate early mathematic abilities, encompassing writing numbers to dictation, comparing visual number symbols, and solving basic arithmetic problems. It complements the existing studies based on math school readiness of preschool children (Blair and Razza, 2007; Duncan et al., 2007; Pagani et al., 2010), by providing a test that can be administered by those teachers and/or health professionals that are accompanying the children throughout the two first years of elementary school. Since the MSR test has proven to be an efficient predictor of children’s proficiency in classical math tests administered 1 year later, it can be used to detect children who are at risk of performing low in mathematics. Empirically validated curricula and specialized neuro-psychological diagnostics and interventions can then be applied depending on the child’s ability level (Wilson et al., 2006; Butterworth and Laurillard, 2010; Clements and Sarama, 2011; Kucian et al., 2011; Skwarchuk et al., 2014; Iuculano et al., 2015).

## Ethics Statement

The present research has obtained the full consent of the Institutional Ethics Committee of the Université Catholique de Louvain (Belgium).

## Author Contributions

SM and CS conceived the test and wrote the manuscript. SM carried out the experiments. CM performed the analysis and designed the figures. All authors discussed the results and contributed to the final manuscript.

## Conflict of Interest Statement

The authors declare that the research was conducted in the absence of any commercial or financial relationships that could be construed as a potential conflict of interest.

## Footnotes

1 This socio-economic index was established in Belgium in 1998 to allocate resources within the framework of the positive discrimination. It is updated every 5 years and it is constructed from the variables “per capita income, educational attainment, unemployment, occupational and comfort level of housing.” To each student corresponds an index defined by its area of residence. It is the smallest administrative unit for which socioeconomic data are available. The socio-economic index is then defined based on the average of the indices of its student population; it does not correspond directly to the area of implantation, or a measure of school performance. It allows one to rank schools on a scale of 1–20, from the lowest socio-economic index to the highest. The choice of variables, indices and formula has been approved by the Government of the French Community (de Villers and Desagher, 2011; Mejias and Schiltz, 2013).

## Supplementary Material

DATA SHEET S1 All novel test material used and data collected in the present study in order to build the “mathematical school readiness test”.

APPENDIX A Inital version of the “mathematical school readiness test”; only 3 items are included in the final version of the test.
