# Supplementary material for: Assessing Mathematical School Readiness
Source: Front Psychol. 2019 May 24;10:1173. doi: 10.3389/fpsyg.2019.01173 (PMC6543806; doi:10.3389/fpsyg.2019.01173)
Supplement: DATA SHEET S1 — All novel test material used and data collected in the present study in order to build the “mathematical school readiness test”. [file Data_Sheet_1.PDF]

MSR_data

id	gender_male1	age_weeks	identify_3	identify_6	identify_8	identify_9	identify_a	identify_f	identify_@	identify_$	identify_sum	writing_4	writing_7	writing_1	writing_6	writing_5	writing_10	writing_9	writing_11	writing_13	writing_16	writing_sum	writing_pomp	comparing_45	comparing_87	comparing_93	comparing_1611	comparing_1314
4	2	70	1	1	1	1	1	1	1	1	8	1	1	1	1	1	0	0	0	0	0	5	0.5	1	0	0	1	0
5	1	78	1	1	1	1	1	1	1	1	8	1	1	1	1	1	1	1	1	0	0	8	0.8	1	1	1	0	1
6	2	72	1	1	1	1	1	1	1	1	8	1	1	1	1	1	1	1	1	0	0	8	0.8	1	1	1	1	1
7	2	72	1	1	1	1	1	1	1	1	8	1	1	1	1	1	1	1	1	1	0	9	0.9	1	1	1	1	1
9	1	80	1	1	1	1	1	1	1	1	8	1	1	1	1	1	1	1	1	1	1	10	1	1	1	1	1	1
11	2	82	1	1	1	1	1	1	1	1	8	1	1	1	1	1	1	1	1	1	1	10	1	1	1	1	1	1
12	2	80	1	1	1	1	1	1	1	1	8	1	1	1	1	1	1	1	1	0	0	8	0.8	1	1	1	1	1
14	2	79	1	1	1	1	1	1	1	1	8	1	1	1	1	1	1	1	1	0	0	8	0.8	1	1	1	1	0
15	1	76	1	1	1	1	1	1	1	1	8	1	1	1	1	1	1	1	1	1	0	9	0.9	1	1	1	1	0
16	2	76	1	1	1	1	1	1	1	1	8	1	1	1	1	1	1	1	1	0	0	8	0.8	1	0	1	1	0
17	2	71	1	1	1	1	1	1	1	1	8	1	1	1	0	1	1	1	1	0	0	7	0.7	1	0	1	1	1
18	2	75	1	1	1	1	1	1	1	1	8	1	0	1	1	1	0	1	1	0	0	6	0.6	1	1	1	0	1
19	2	76	1	1	1	1	1	0	1	1	7	1	1	1	1	1	1	1	1	0	0	8	0.8	1	0	1	1	1
20	2	72	1	1	1	1	1	1	1	1	8	1	1	1	1	1	1	1	1	1	0	9	0.9	1	1	1	1	1
21	1	75	1	1	1	1	1	1	1	1	8	1	1	1	1	1	1	1	1	0	0	8	0.8	1	1	1	1	1
22	1	70	1	1	1	1	1	1	1	1	8	1	1	1	1	1	1	1	1	1	1	10	1	1	1	1	1	1
23	1	74	1	1	1	1	1	1	1	1	8	1	1	1	1	1	1	1	1	0	0	8	0.8	1	1	1	1	1
25	2	75	1	1	1	1	1	1	1	1	8	1	1	1	1	1	1	1	1	0	0	8	0.8	1	0	1	0	0
27	1	76	1	1	1	1	1	1	1	1	8	1	1	1	1	1	1	1	1	0	0	8	0.8	1	0	1	0	0
28	2	77	1	1	1	1	1	1	1	1	8	1	1	1	1	1	1	1	1	1	1	10	1	0	1	1	0	0
29	1	76	1	1	1	1	1	1	1	1	8	1	1	1	1	1	1	1	1	0	0	8	0.8	1	1	1	1	1
30	2	75	1	1	1	1	1	1	1	1	8	1	1	1	1	1	1	1	1	1	0	9	0.9	1	1	1	1	1
31	2	70	1	1	1	1	1	1	1	1	8	1	1	1	1	1	1	1	1	0	1	9	0.9	1	1	1	1	1
32	2	71	1	1	1	1	1	1	1	1	8	1	1	1	1	1	1	1	1	1	0	9	0.9	1	0	1	1	1
33	2	81	1	1	1	1	1	1	1	1	8	1	1	1	1	1	1	1	1	1	1	10	1	1	1	1	1	1
34	2	77	1	1	1	1	1	1	1	1	8	1	1	1	1	1	1	1	1	1	1	10	1	1	1	1	1	1
35	1	76	1	1	1	1	1	1	1	1	8	1	1	1	1	1	1	1	1	1	0	9	0.9	1	1	1	1	1
37	1	69	1	1	1	1	1	1	1	1	8	1	1	1	1	1	1	1	1	1	1	10	1	1	1	1	1	1
38	1	72	1	1	1	1	1	1	1	1	8	1	1	1	1	1	1	1	1	1	1	10	1	1	1	1	1	1
39	2	74	1	1	1	1	1	1	1	1	8	1	1	1	1	1	1	1	1	1	1	10	1	1	1	1	1	1
40	1	80	1	1	1	1	1	1	1	1	8	1	1	1	1	1	1	1	1	1	0	9	0.9	1	1	1	1	1
41	1	74	1	1	1	1	1	1	1	1	8	1	1	1	1	1	1	1	1	1	1	10	1	1	1	1	1	1
43	1	72	1	1	1	1	1	1	1	1	8	1	1	1	1	1	1	1	1	1	1	10	1	1	1	1	1	1
45	2	81	1	1	1	1	1	1	1	1	8	1	1	1	0	1	0	0	0	0	0	4	0.4	1	0	1	1	1
46	2	69	1	1	1	1	1	0	1	0	6	1	1	1	1	1	1	0	0	0	0	6	0.6	1	1	1	1	1
48	1	74	1	1	1	1	1	1	1	1	8	1	1	1	1	1	1	1	1	0	0	8	0.8	1	1	1	1	1
49	2	80	1	1	1	1	1	1	1	0	7	1	1	1	1	1	1	1	1	0	0	8	0.8	1	1	1	1	0
51	1	80	1	1	1	1	1	1	1	1	8	1	1	1	1	1	1	1	1	1	1	10	1	1	1	1	1	1
52	1	74	1	1	1	1	1	1	1	1	8	1	0	1	1	1	0	1	0	0	0	5	0.5	1	1	1	1	1
55	2	71	1	1	1	1	1	1	1	1	8	1	1	1	1	1	1	1	1	0	0	8	0.8	1	1	1	0	0
57	2	79	1	1	1	1	1	1	1	1	8	1	1	1	1	1	1	1	1	1	1	10	1	1	1	1	1	1
58	2	80	1	1	1	1	0	0	0	1	5	1	1	1	1	1	1	1	1	1	1	10	1	1	1	1	1	1
59	1	75	1	1	1	1	1	1	1	1	8	1	1	1	1	1	1	1	1	1	1	10	1	1	1	1	1	1
60	2	73	1	1	1	1	1	1	0	1	7	1	1	1	1	1	1	1	1	1	0	9	0.9	1	1	1	1	1
61	2	75	1	1	1	1	1	1	1	1	8	1	1	1	1	1	1	0	1	1	0	8	0.8	1	1	1	1	1
62	1	71	1	1	1	1	1	1	1	1	8	1	1	1	1	1	1	1	1	1	0	9	0.9	1	1	1	1	1
63	2	70	1	1	1	1	1	1	0	0	6	1	1	1	1	1	1	1	1	1	0	9	0.9	1	1	1	1	1
64	1	75	1	1	1	1	1	1	1	1	8	1	0	1	1	1	1	0	1	0	0	6	0.6	1	1	1	1	0
65	2	77	1	1	1	1	1	1	1	0	7	1	1	1	0	1	0	0	0	0	0	4	0.4	1	0	0	0	1
66	1	81	1	1	1	1	1	1	1	1	8	1	1	1	1	1	1	1	1	0	0	8	0.8	0	0	0	1	0
67	2	71	1	1	1	1	1	1	1	1	8	1	1	1	1	1	1	1	1	0	0	8	0.8	0	0	1	0	0
68	1	71	1	1	1	1	1	1	1	1	8	1	1	1	1	1	1	1	0	0	0	7	0.7	1	1	1	1	1
69	2	72	1	1	1	1	1	1	1	1	8	1	1	1	1	1	1	1	0	0	0	7	0.7	1	1	1	0	0
70	1	76	1	1	1	1	1	1	1	1	8	1	1	1	1	1	1	1	1	0	1	9	0.9	1	1	1	1	1
71	2	75	1	1	1	1	1	1	1	1	8	1	1	1	1	1	1	1	1	0	0	8	0.8	1	1	1	0	1
72	1	69	1	1	1	1	1	1	1	1	8	1	1	1	1	1	1	1	1	1	1	10	1	1	1	1	1	1
73	2	77	1	1	1	1	1	1	1	1	8	1	1	1	1	1	1	1	0	0	0	7	0.7	1	1	1	0	1
74	1	82	1	1	1	1	0	1	1	1	7	1	1	1	1	1	1	1	1	0	0	8	0.8	1	1	1	1	0
75	1	77	1	1	1	1	1	1	1	1	8	1	1	1	1	1	1	1	1	0	0	8	0.8	1	1	1	1	1
76	2	75	1	1	1	1	1	1	1	1	8	1	1	1	1	1	1	1	1	0	0	8	0.8	1	1	1	1	1
77	1	76	1	1	1	1	1	1	1	1	8	1	1	1	1	1	1	1	1	1	1	10	1	1	1	1	1	1
78	2	75	1	1	1	1	1	1	1	1	8	1	1	1	1	1	1	1	1	1	1	10	1	1	1	1	1	1
79	2	73	1	1	1	1	1	1	1	1	8	1	1	1	1	1	1	1	1	0	0	8	0.8	1	1	1	1	1
80	2	80	1	1	1	1	1	1	1	1	8	1	1	1	1	1	1	1	1	1	1	10	1	1	1	1	1	1
81	1	76	1	1	1	1	1	1	1	1	8	1	1	1	1	1	1	1	1	1	1	10	1	1	1	1	1	1
82	1	79	1	1	1	1	1	1	1	1	8	1	1	1	1	1	1	1	1	1	1	10	1	1	1	1	1	1
83	2	69	1	1	1	1	1	1	1	1	8	1	1	1	1	1	1	1	1	0	0	8	0.8	1	1	1	1	1
84	1	80	1	1	1	1	1	1	1	1	8	1	1	1	1	1	1	1	1	0	0	8	0.8	0	1	1	1	1

85	2	77	1	1	1	1	1	1	1	1	8	1	1	1	1	1	1	1	1	1	1	10	1	1	1	1	1	1
86	2	78	1	1	1	1	1	1	1	1	8	1	1	1	1	1	1	1	1	1	1	10	1	1	1	1	1	1
87	2	80	1	1	1	1	1	1	1	1	8	1	1	1	1	1	1	1	1	0	1	9	0.9	1	0	1	1	0
88	2	81	1	1	1	1	1	1	1	1	8	1	1	1	1	1	1	1	1	1	0	9	0.9	1	1	1	0	0
89	1	76	1	1	1	1	0	1	1	1	7	1	1	1	1	1	1	1	1	0	0	8	0.8	1	1	1	1	1
90	1	70	1	1	1	1	1	1	1	1	8	1	1	1	1	1	1	1	1	1	1	10	1	1	1	1	1	1
91	1	77	1	1	1	1	1	1	1	1	8	1	1	1	1	1	1	1	1	1	1	10	1	1	1	1	1	1
92	2	70	1	1	1	1	1	1	1	1	8	1	1	1	1	1	1	1	1	1	1	10	1	1	1	1	1	1
93	2	77	1	1	1	1	1	1	1	1	8	1	1	1	1	1	1	1	1	1	1	10	1	1	1	1	1	1
94	1	70	1	1	1	1	1	1	1	1	8	1	1	1	1	1	1	1	1	1	1	10	1	1	1	1	1	1
95	1	80	1	1	1	1	1	1	1	1	8	1	1	1	1	1	1	1	1	1	1	10	1	1	1	1	1	1
97	2	70	1	1	1	1	1	1	1	1	8	1	1	1	1	1	1	1	1	1	0	9	0.9	1	1	1	1	1
98	1	83	1	1	1	1	1	1	1	1	8	1	1	1	1	1	1	1	1	1	0	9	0.9	1	1	1	1	1
99	2	78	1	1	1	1	1	1	1	1	8	1	1	1	1	1	1	1	1	0	0	8	0.8	1	1	1	1	1
100	1	70	1	1	1	1	1	1	1	0	7	1	1	1	1	1	1	1	1	1	0	9	0.9	1	1	1	1	1
101	2	79	1	1	1	1	1	1	1	1	8	1	1	1	1	1	0	1	0	0	1	7	0.7	1	0	1	1	0
102	2	75	1	1	1	1	1	1	0	0	6	1	1	1	1	1	1	1	1	1	0	9	0.9	0	1	1	1	1
103	1	79	1	1	1	1	1	1	1	1	8	1	1	1	1	1	1	1	1	0	1	9	0.9	1	1	1	1	1
104	2	78	1	1	1	1	1	1	1	1	8	1	1	1	1	1	1	1	1	1	0	9	0.9	1	1	1	1	1
105	1	75	1	1	1	1	1	1	1	1	8	1	1	1	1	1	1	1	1	1	1	10	1	1	1	1	1	1
106	2	80	1	1	1	1	1	1	1	1	8	1	1	1	1	1	1	1	1	1	1	10	1	1	1	1	1	1
107	1	75	1	1	1	1	1	1	1	1	8	1	1	1	1	1	1	1	1	1	1	10	1	1	1	1	1	1
108	2	73	1	1	1	1	0	1	0	0	5	1	1	1	0	1	1	0	1	1	1	8	0.8	1	1	1	1	1
109	1	74	1	1	1	1	1	1	0	1	7	1	1	1	1	1	1	1	1	1	0	9	0.9	0	1	1	0	0
110	2	77	1	1	1	1	1	1	1	1	8	1	1	1	1	1	1	1	1	0	1	9	0.9	1	0	0	1	1
111	1	75	1	1	1	1	1	1	1	1	8	1	1	1	1	1	1	1	1	1	0	9	0.9	1	1	1	1	1
112	2	71	1	1	1	1	1	1	1	1	8	1	1	1	1	1	1	1	1	1	0	9	0.9	1	1	1	1	0
113	2	70	1	1	1	1	1	1	1	1	8	1	1	1	1	1	0	1	1	1	0	8	0.8	0	0	1	1	1
114	1	79	1	1	1	1	1	1	1	1	8	1	1	1	1	1	1	1	1	1	1	10	1	1	1	1	1	1
115	1	80	1	1	1	1	1	1	1	1	8	1	1	1	1	1	1	1	1	0	0	8	0.8	1	1	1	1	0
116	2	78	1	1	1	1	1	1	1	1	8	1	1	1	1	1	1	1	1	1	1	10	1	1	1	1	1	1
117	1	79	1	1	1	1	1	1	1	1	8	1	1	1	1	1	1	1	1	1	1	10	1	1	1	1	1	1
118	1	76	1	1	1	1	1	1	1	1	8	1	1	1	1	1	1	1	1	1	1	10	1	1	1	1	1	1
119	2	75	1	1	1	1	1	1	1	1	8	1	1	1	1	1	1	1	1	1	1	10	1	1	1	1	1	1
120	2	77	1	1	1	1	1	1	1	1	8	1	1	1	1	1	1	1	1	1	0	9	0.9	1	1	1	1	1
121	2	78	1	1	1	1	1	1	1	1	8	1	1	1	1	1	1	1	1	0	0	8	0.8	1	1	1	1	1
122	1	72	1	1	1	1	1	1	1	1	8	1	1	1	1	1	1	1	1	1	1	10	1	1	1	1	1	1
123	2	74	1	1	1	1	1	1	1	1	8	1	1	1	1	1	1	1	1	0	0	8	0.8	1	1	1	1	1
124	1	78	1	1	1	1	1	1	1	1	8	1	1	1	1	1	1	1	1	1	1	10	1	1	1	1	1	1
125	2	72	1	1	1	1	1	1	1	1	8	1	1	1	1	1	1	1	1	0	0	8	0.8	1	1	1	0	1
126	2	73	1	1	1	1	1	1	1	1	8	1	1	1	1	1	1	1	1	0	0	8	0.8	1	1	1	0	0
127	1	72	1	1	1	1	1	1	1	1	8	1	1	1	1	1	1	1	1	1	0	9	0.9	1	1	1	1	1
128	2	78	1	1	1	1	1	1	1	1	8	1	1	1	1	1	1	1	1	1	1	10	1	1	1	1	1	1
129	1	71	1	1	1	1	1	1	1	1	8	1	0	1	1	1	0	1	1	0	0	6	0.6	1	0	1	1	1
130	1	77	1	1	1	1	1	1	1	1	8	1	1	1	1	1	1	1	1	1	1	10	1	1	1	1	1	1
131	1	79	1	1	1	1	1	1	1	1	8	1	1	1	1	1	1	1	1	0	0	8	0.8	1	1	1	1	1
132	2	80	1	1	1	1	1	1	1	1	8	1	1	1	1	1	1	1	1	0	0	8	0.8	1	1	1	1	1
133	1	78	1	1	1	1	1	1	1	1	8	1	1	1	1	1	1	1	1	0	0	8	0.8	1	1	1	1	1
134	1	72	1	1	1	1	1	1	1	1	8	1	1	1	1	1	1	0	1	0	0	7	0.7	1	1	1	1	0
135	1	72	1	1	1	1	1	1	1	1	8	1	1	1	1	1	1	1	1	1	0	9	0.9	1	1	1	1	1
136	2	72	1	1	1	1	1	1	1	1	8	1	1	1	1	1	0	1	1	1	1	9	0.9	1	1	1	1	1
137	2	86	1	1	1	1	1	1	1	1	8	1	1	1	1	1	1	1	1	0	0	8	0.8	1	1	1	1	1
138	2	74	1	1	1	1	1	1	1	1	8	1	1	1	1	1	1	1	1	1	0	9	0.9	1	1	1	1	1
139	1	73	1	1	1	1	0	0	1	1	6	1	1	1	1	1	1	1	1	0	0	8	0.8	1	1	1	1	1
140	2	75	1	1	1	1	1	1	1	1	8	1	1	1	1	1	0	0	1	1	0	7	0.7	1	1	1	1	1
142	2	73	.	.	.	.	.	.	.	.	0	.	.	.	.	.	.	.	.	.	.	8	0.8	.	.	.	.	.
143	1	80	.	.	.	.	.	.	.	.	0	.	.	.	.	.	.	.	.	.	.	10	1	.	.	.	.	.
144	1	85	.	.	.	.	.	.	.	.	0	.	.	.	.	.	.	.	.	.	.	8	0.8	.	.	.	.	.
145	2	82	.	.	.	.	.	.	.	.	0	.	.	.	.	.	.	.	.	.	.	9	0.9	.	.	.	.	.
146	1	76	.	.	.	.	.	.	.	.	0	.	.	.	.	.	.	.	.	.	.	5	0.5	.	.	.	.	.
147	1	86	.	.	.	.	.	.	.	.	0	.	.	.	.	.	.	.	.	.	.	10	1	.	.	.	.	.
148	2	76	.	.	.	.	.	.	.	.	0	.	.	.	.	.	.	.	.	.	.	3	0.3	.	.	.	.	.
149	2	69	.	.	.	.	.	.	.	.	0	.	.	.	.	.	.	.	.	.	.	9	0.9	.	.	.	.	.
150	1	76	.	.	.	.	.	.	.	.	7	.	.	.	.	.	.	.	.	.	.	5	0.5	.	.	.	.	.
151	1	75	.	.	.	.	.	.	.	.	0	.	.	.	.	.	.	.	.	.	.	9	0.9	.	.	.	.	.
152	2	70	.	.	.	.	.	.	.	.	0	.	.	.	.	.	.	.	.	.	.	8	0.8	.	.	.	.	.
154	1	70	.	.	.	.	.	.	.	.	0	.	.	.	.	.	.	.	.	.	.	9	0.9	.	.	.	.	.
155	2	73	.	.	.	.	.	.	.	.	0	.	.	.	.	.	.	.	.	.	.	9	0.9	.	.	.	.	.
157	2	80	.	.	.	.	.	.	.	.	0	.	.	.	.	.	.	.	.	.	.	7	0.7	.	.	.	.	.

160	2	76	.	.	.	.	.	.	.	.	0	.	.	.	.	.	.	.	.	.	.	8	0.8	.	.	.	.	.
161	2	75	.	.	.	.	.	.	.	.	7	.	.	.	.	.	.	.	.	.	.	7	0.7	.	.	.	.	.
162	2	78	.	.	.	.	.	.	.	.	0	.	.	.	.	.	.	.	.	.	.	8	0.8	.	.	.	.	.
165	2	74	.	.	.	.	.	.	.	.	6	.	.	.	.	.	.	.	.	.	.	8	0.8	.	.	.	.	.
167	2	72	.	.	.	.	.	.	.	.	0	.	.	.	.	.	.	.	.	.	.	8	0.8	.	.	.	.	.
168	2	78	.	.	.	.	.	.	.	.	0	.	.	.	.	.	.	.	.	.	.	8	0.8	.	.	.	.	.
169	2	71	.	.	.	.	.	.	.	.	0	.	.	.	.	.	.	.	.	.	.	8	0.8	.	.	.	.	.
170	1	75	.	.	.	.	.	.	.	.	7	.	.	.	.	.	.	.	.	.	.	6	0.6	.	.	.	.	.
171	1	70	.	.	.	.	.	.	.	.	6	.	.	.	.	.	.	.	.	.	.	8	0.8	.	.	.	.	.
172	2	80	.	.	.	.	.	.	.	.	6	.	.	.	.	.	.	.	.	.	.	8	0.8	.	.	.	.	.
173	1	78	.	.	.	.	.	.	.	.	0	.	.	.	.	.	.	.	.	.	.	10	1	.	.	.	.	.
175	2	71	.	.	.	.	.	.	.	.	0	.	.	.	.	.	.	.	.	.	.	7	0.7	.	.	.	.	.
178	2	80	.	.	.	.	.	.	.	.	3	.	.	.	.	.	.	.	.	.	.	8	0.8	.	.	.	.	.
180	1	74	.	.	.	.	.	.	.	.	0	.	.	.	.	.	.	.	.	.	.	9	0.9	.	.	.	.	.
182	1	69	.	.	.	.	.	.	.	.	6	.	.	.	.	.	.	.	.	.	.	9	0.9	.	.	.	.	.
183	1	76	.	.	.	.	.	.	.	.	7	.	.	.	.	.	.	.	.	.	.	10	1	.	.	.	.	.
185	2	74	.	.	.	.	.	.	.	.	0	.	.	.	.	.	.	.	.	.	.	8	0.8	.	.	.	.	.
186	2	85	.	.	.	.	.	.	.	.	0	.	.	.	.	.	.	.	.	.	.	8	0.8	.	.	.	.	.
187	2	72	.	.	.	.	.	.	.	.	0	.	.	.	.	.	.	.	.	.	.	9	0.9	.	.	.	.	.
188	1	77	.	.	.	.	.	.	.	.	6	.	.	.	.	.	.	.	.	.	.	7	0.7	.	.	.	.	.
189	2	78	.	.	.	.	.	.	.	.	5	.	.	.	.	.	.	.	.	.	.	8	0.8	.	.	.	.	.
192	1	76	.	.	.	.	.	.	.	.	0	.	.	.	.	.	.	.	.	.	.	7	0.7	.	.	.	.	.
194	2	95	.	.	.	.	.	.	.	.	5	.	.	.	.	.	.	.	.	.	.	8	0.8	.	.	.	.	.
195	1	71	.	.	.	.	.	.	.	.	0	.	.	.	.	.	.	.	.	.	.	8	0.8	.	.	.	.	.
196	2	74	.	.	.	.	.	.	.	.	0	.	.	.	.	.	.	.	.	.	.	1	0.1	.	.	.	.	.
198	2	70	.	.	.	.	.	.	.	.	0	.	.	.	.	.	.	.	.	.	.	8	0.8	.	.	.	.	.
199	1	91	.	.	.	.	.	.	.	.	7	.	.	.	.	.	.	.	.	.	.	8	0.8	.	.	.	.	.
201	1	72	.	.	.	.	.	.	.	.	0	.	.	.	.	.	.	.	.	.	.	6	0.6	.	.	.	.	.
202	2	79	.	.	.	.	.	.	.	.	0	.	.	.	.	.	.	.	.	.	.	10	1	.	.	.	.	.
203	2	76	.	.	.	.	.	.	.	.	0	.	.	.	.	.	.	.	.	.	.	9	0.9	.	.	.	.	.
204	1	70	.	.	.	.	.	.	.	.	0	.	.	.	.	.	.	.	.	.	.	10	1	.	.	.	.	.
205	2	95	.	.	.	.	.	.	.	.	0	.	.	.	.	.	.	.	.	.	.	10	1	.	.	.	.	.
206	1	81	.	.	.	.	.	.	.	.	0	.	.	.	.	.	.	.	.	.	.	10	1	.	.	.	.	.
207	2	72	.	.	.	.	.	.	.	.	0	.	.	.	.	.	.	.	.	.	.	8	0.8	.	.	.	.	.
208	1	69	.	.	.	.	.	.	.	.	0	.	.	.	.	.	.	.	.	.	.	8	0.8	.	.	.	.	.
209	1	71	.	.	.	.	.	.	.	.	2	.	.	.	.	.	.	.	.	.	.	9	0.9	.	.	.	.	.
210	2	78	.	.	.	.	.	.	.	.	0	.	.	.	.	.	.	.	.	.	.	10	1	.	.	.	.	.
211	2	80	.	.	.	.	.	.	.	.	0	.	.	.	.	.	.	.	.	.	.	10	1	.	.	.	.	.
212	1	71	.	.	.	.	.	.	.	.	0	.	.	.	.	.	.	.	.	.	.	10	1	.	.	.	.	.
215	1	78	.	.	.	.	.	.	.	.	0	.	.	.	.	.	.	.	.	.	.	10	1	.	.	.	.	.
216	2	74	.	.	.	.	.	.	.	.	0	.	.	.	.	.	.	.	.	.	.	10	1	.	.	.	.	.
217	1	75	.	.	.	.	.	.	.	.	0	.	.	.	.	.	.	.	.	.	.	7	0.7	.	.	.	.	.
219	2	76	.	.	.	.	.	.	.	.	0	.	.	.	.	.	.	.	.	.	.	10	1	.	.	.	.	.
221	2	75	.	.	.	.	.	.	.	.	0	.	.	.	.	.	.	.	.	.	.	7	0.7	.	.	.	.	.
222	1	80	.	.	.	.	.	.	.	.	0	.	.	.	.	.	.	.	.	.	.	10	1	.	.	.	.	.
223	2	87	.	.	.	.	.	.	.	.	0	.	.	.	.	.	.	.	.	.	.	10	1	.	.	.	.	.
224	2	69	.	.	.	.	.	.	.	.	0	.	.	.	.	.	.	.	.	.	.	10	1	.	.	.	.	.
229	1	79	.	.	.	.	.	.	.	.	4	.	.	.	.	.	.	.	.	.	.	3	0.3	.	.	.	.	.
230	1	79	.	.	.	.	.	.	.	.	0	.	.	.	.	.	.	.	.	.	.	10	1	.	.	.	.	.
231	2	77	.	.	.	.	.	.	.	.	0	.	.	.	.	.	.	.	.	.	.	9	0.9	.	.	.	.	.
232	2	74	.	.	.	.	.	.	.	.	7	.	.	.	.	.	.	.	.	.	.	8	0.8	.	.	.	.	.
233	2	72	.	.	.	.	.	.	.	.	0	.	.	.	.	.	.	.	.	.	.	7	0.7	.	.	.	.	.
234	2	73	.	.	.	.	.	.	.	.	0	.	.	.	.	.	.	.	.	.	.	8	0.8	.	.	.	.	.
235	1	78	.	.	.	.	.	.	.	.	0	.	.	.	.	.	.	.	.	.	.	9	0.9	.	.	.	.	.
236	2	78	.	.	.	.	.	.	.	.	0	.	.	.	.	.	.	.	.	.	.	7	0.7	.	.	.	.	.
237	1	71	.	.	.	.	.	.	.	.	4	.	.	.	.	.	.	.	.	.	.	2	0.2	.	.	.	.	.
238	2	75	.	.	.	.	.	.	.	.	0	.	.	.	.	.	.	.	.	.	.	7	0.7	.	.	.	.	.
239	1	83	.	.	.	.	.	.	.	.	0	.	.	.	.	.	.	.	.	.	.	9	0.9	.	.	.	.	.
240	2	89	.	.	.	.	.	.	.	.	0	.	.	.	.	.	.	.	.	.	.	9	0.9	.	.	.	.	.
242	2	71	.	.	.	.	.	.	.	.	0	.	.	.	.	.	.	.	.	.	.	10	1	.	.	.	.	.
243	1	78	.	.	.	.	.	.	.	.	7	.	.	.	.	.	.	.	.	.	.	8	0.8	.	.	.	.	.
244	1	80	.	.	.	.	.	.	.	.	0	.	.	.	.	.	.	.	.	.	.	10	1	.	.	.	.	.
245	1	71	.	.	.	.	.	.	.	.	0	.	.	.	.	.	.	.	.	.	.	10	1	.	.	.	.	.
246	2	80	.	.	.	.	.	.	.	.	0	.	.	.	.	.	.	.	.	.	.	10	1	.	.	.	.	.
247	1	74	.	.	.	.	.	.	.	.	0	.	.	.	.	.	.	.	.	.	.	10	1	.	.	.	.	.
248	2	78	.	.	.	.	.	.	.	.	0	.	.	.	.	.	.	.	.	.	.	9	0.9	.	.	.	.	.
249	1	75	.	.	.	.	.	.	.	.	0	.	.	.	.	.	.	.	.	.	.	10	1	.	.	.	.	.
250	1	74	.	.	.	.	.	.	.	.	0	.	.	.	.	.	.	.	.	.	.	8	0.8	.	.	.	.	.
251	2	80	.	.	.	.	.	.	.	.	0	.	.	.	.	.	.	.	.	.	.	8	0.8	.	.	.	.	.
253	2	75	.	.	.	.	.	.	.	.	0	.	.	.	.	.	.	.	.	.	.	9	0.9	.	.	.	.	.

254	1	75	.	.	.	.	.	.	.	.	0	.	.	.	.	.	.	.	.	.	.	7	0.7	.	.	.	.	.
255	2	75	.	.	.	.	.	.	.	.	0	.	.	.	.	.	.	.	.	.	.	9	0.9	.	.	.	.	.
256	2	81	.	.	.	.	.	.	.	.	0	.	.	.	.	.	.	.	.	.	.	10	1	.	.	.	.	.
257	2	75	.	.	.	.	.	.	.	.	0	.	.	.	.	.	.	.	.	.	.	8	0.8	.	.	.	.	.
258	1	74	.	.	.	.	.	.	.	.	0	.	.	.	.	.	.	.	.	.	.	9	0.9	.	.	.	.	.
259	1	78	.	.	.	.	.	.	.	.	0	.	.	.	.	.	.	.	.	.	.	10	1	.	.	.	.	.
260	2	77	.	.	.	.	.	.	.	.	0	.	.	.	.	.	.	.	.	.	.	7	0.7	.	.	.	.	.
261	1	71	.	.	.	.	.	.	.	.	4	.	.	.	.	.	.	.	.	.	.	7	0.7	.	.	.	.	.
262	2	74	.	.	.	.	.	.	.	.	0	.	.	.	.	.	.	.	.	.	.	10	1	.	.	.	.	.
263	2	73	.	.	.	.	.	.	.	.	4	.	.	.	.	.	.	.	.	.	.	10	1	.	.	.	.	.
264	1	78	.	.	.	.	.	.	.	.	0	.	.	.	.	.	.	.	.	.	.	10	1	.	.	.	.	.
266	1	74	.	.	.	.	.	.	.	.	0	.	.	.	.	.	.	.	.	.	.	10	1	.	.	.	.	.
268	1	77	.	.	.	.	.	.	.	.	0	.	.	.	.	.	.	.	.	.	.	9	0.9	.	.	.	.	.
269	1	79	.	.	.	.	.	.	.	.	0	.	.	.	.	.	.	.	.	.	.	10	1	.	.	.	.	.
270	1	79	.	.	.	.	.	.	.	.	0	.	.	.	.	.	.	.	.	.	.	9	0.9	.	.	.	.	.
271	1	75	.	.	.	.	.	.	.	.	0	.	.	.	.	.	.	.	.	.	.	10	1	.	.	.	.	.
272	1	80	.	.	.	.	.	.	.	.	0	.	.	.	.	.	.	.	.	.	.	10	1	.	.	.	.	.
273	1	73	.	.	.	.	.	.	.	.	0	.	.	.	.	.	.	.	.	.	.	10	1	.	.	.	.	.
274	1	70	.	.	.	.	.	.	.	.	0	.	.	.	.	.	.	.	.	.	.	10	1	.	.	.	.	.
275	2	74	.	.	.	.	.	.	.	.	0	.	.	.	.	.	.	.	.	.	.	10	1	.	.	.	.	.
276	2	73	.	.	.	.	.	.	.	.	0	.	.	.	.	.	.	.	.	.	.	10	1	.	.	.	.	.
277	2	74	.	.	.	.	.	.	.	.	0	.	.	.	.	.	.	.	.	.	.	9	0.9	.	.	.	.	.
278	2	75	.	.	.	.	.	.	.	.	0	.	.	.	.	.	.	.	.	.	.	10	1	.	.	.	.	.
279	2	79	.	.	.	.	.	.	.	.	0	.	.	.	.	.	.	.	.	.	.	10	1	.	.	.	.	.
280	1	81	.	.	.	.	.	.	.	.	7	.	.	.	.	.	.	.	.	.	.	7	0.7	.	.	.	.	.
281	2	79	.	.	.	.	.	.	.	.	0	.	.	.	.	.	.	.	.	.	.	10	1	.	.	.	.	.
282	1	74	.	.	.	.	.	.	.	.	7	.	.	.	.	.	.	.	.	.	.	10	1	.	.	.	.	.
283	2	80	.	.	.	.	.	.	.	.	0	.	.	.	.	.	.	.	.	.	.	8	0.8	.	.	.	.	.
284	2	79	.	.	.	.	.	.	.	.	0	.	.	.	.	.	.	.	.	.	.	9	0.9	.	.	.	.	.
285	1	79	.	.	.	.	.	.	.	.	0	.	.	.	.	.	.	.	.	.	.	9	0.9	.	.	.	.	.
286	1	77	.	.	.	.	.	.	.	.	0	.	.	.	.	.	.	.	.	.	.	8	0.8	.	.	.	.	.
287	1	70	.	.	.	.	.	.	.	.	0	.	.	.	.	.	.	.	.	.	.	8	0.8	.	.	.	.	.
288	2	71	.	.	.	.	.	.	.	.	6	.	.	.	.	.	.	.	.	.	.	10	1	.	.	.	.	.
289	2	71	.	.	.	.	.	.	.	.	0	.	.	.	.	.	.	.	.	.	.	7	0.7	.	.	.	.	.
291	1	75	.	.	.	.	.	.	.	.	0	.	.	.	.	.	.	.	.	.	.	8	0.8	.	.	.	.	.
292	1	83	.	.	.	.	.	.	.	.	0	.	.	.	.	.	.	.	.	.	.	9	0.9	.	.	.	.	.
294	2	75	.	.	.	.	.	.	.	.	5	.	.	.	.	.	.	.	.	.	.	8	0.8	.	.	.	.	.
295	1	75	.	.	.	.	.	.	.	.	4	.	.	.	.	.	.	.	.	.	.	9	0.9	.	.	.	.	.
296	1	74	.	.	.	.	.	.	.	.	0	.	.	.	.	.	.	.	.	.	.	6	0.6	.	.	.	.	.
297	2	79	.	.	.	.	.	.	.	.	7	.	.	.	.	.	.	.	.	.	.	6	0.6	.	.	.	.	.
298	1	77	.	.	.	.	.	.	.	.	7	.	.	.	.	.	.	.	.	.	.	8	0.8	.	.	.	.	.
299	2	72	.	.	.	.	.	.	.	.	5	.	.	.	.	.	.	.	.	.	.	6	0.6	.	.	.	.	.
300	2	76	.	.	.	.	.	.	.	.	0	.	.	.	.	.	.	.	.	.	.	10	1	.	.	.	.	.
301	1	72	.	.	.	.	.	.	.	.	0	.	.	.	.	.	.	.	.	.	.	8	0.8	.	.	.	.	.
302	2	76	.	.	.	.	.	.	.	.	0	.	.	.	.	.	.	.	.	.	.	8	0.8	.	.	.	.	.
303	1	75	.	.	.	.	.	.	.	.	0	.	.	.	.	.	.	.	.	.	.	9	0.9	.	.	.	.	.
304	2	73	.	.	.	.	.	.	.	.	4	.	.	.	.	.	.	.	.	.	.	8	0.8	.	.	.	.	.
306	1	76	.	.	.	.	.	.	.	.	4	.	.	.	.	.	.	.	.	.	.	10	1	.	.	.	.	.
307	2	80	.	.	.	.	.	.	.	.	0	.	.	.	.	.	.	.	.	.	.	10	1	.	.	.	.	.
308	2	69	.	.	.	.	.	.	.	.	0	.	.	.	.	.	.	.	.	.	.	7	0.7	.	.	.	.	.
309	1	71	.	.	.	.	.	.	.	.	0	.	.	.	.	.	.	.	.	.	.	7	0.7	.	.	.	.	.
310	2	88	.	.	.	.	.	.	.	.	0	.	.	.	.	.	.	.	.	.	.	10	1	.	.	.	.	.
311	2	71	.	.	.	.	.	.	.	.	0	.	.	.	.	.	.	.	.	.	.	7	0.7	.	.	.	.	.
312	2	73	.	.	.	.	.	.	.	.	0	.	.	.	.	.	.	.	.	.	.	9	0.9	.	.	.	.	.
313	2	78	.	.	.	.	.	.	.	.	0	.	.	.	.	.	.	.	.	.	.	9	0.9	.	.	.	.	.
315	1	77	.	.	.	.	.	.	.	.	7	.	.	.	.	.	.	.	.	.	.	8	0.8	.	.	.	.	.
316	2	74	.	.	.	.	.	.	.	.	4	.	.	.	.	.	.	.	.	.	.	7	0.7	.	.	.	.	.
317	2	75	.	.	.	.	.	.	.	.	0	.	.	.	.	.	.	.	.	.	.	7	0.7	.	.	.	.	.
318	2	77	.	.	.	.	.	.	.	.	0	.	.	.	.	.	.	.	.	.	.	6	0.6	.	.	.	.	.
319	2	75	.	.	.	.	.	.	.	.	0	.	.	.	.	.	.	.	.	.	.	7	0.7	.	.	.	.	.
320	2	75	.	.	.	.	.	.	.	.	0	.	.	.	.	.	.	.	.	.	.	9	0.9	.	.	.	.	.
322	1	73	.	.	.	.	.	.	.	.	0	.	.	.	.	.	.	.	.	.	.	10	1	.	.	.	.	.
325	2	77	.	.	.	.	.	.	.	.	0	.	.	.	.	.	.	.	.	.	.	10	1	.	.	.	.	.
326	1	74	.	.	.	.	.	.	.	.	2	.	.	.	.	.	.	.	.	.	.	9	0.9	.	.	.	.	.
327	1	76	.	.	.	.	.	.	.	.	0	.	.	.	.	.	.	.	.	.	.	10	1	.	.	.	.	.
329	2	73	.	.	.	.	.	.	.	.	0	.	.	.	.	.	.	.	.	.	.	9	0.9	.	.	.	.	.
330	1	72	.	.	.	.	.	.	.	.	7	.	.	.	.	.	.	.	.	.	.	9	0.9	.	.	.	.	.
331	2	71	.	.	.	.	.	.	.	.	7	.	.	.	.	.	.	.	.	.	.	8	0.8	.	.	.	.	.
332	1	77	.	.	.	.	.	.	.	.	0	.	.	.	.	.	.	.	.	.	.	9	0.9	.	.	.	.	.

333	1	73	.	.	.	.	.	.	.	.	0	.	.	.	.	.	.	.	.	.	.	5	0.5	.	.	.	.	.
334	2	71	.	.	.	.	.	.	.	.	0	.	.	.	.	.	.	.	.	.	.	10	1	.	.	.	.	.
335	1	79	.	.	.	.	.	.	.	.	0	.	.	.	.	.	.	.	.	.	.	10	1	.	.	.	.	.
336	1	70	.	.	.	.	.	.	.	.	0	.	.	.	.	.	.	.	.	.	.	8	0.8	.	.	.	.	.
337	1	80	.	.	.	.	.	.	.	.	0	.	.	.	.	.	.	.	.	.	.	10	1	.	.	.	.	.
339	1	77	.	.	.	.	.	.	.	.	0	.	.	.	.	.	.	.	.	.	.	10	1	.	.	.	.	.
340	2	79	.	.	.	.	.	.	.	.	7	.	.	.	.	.	.	.	.	.	.	10	1	.	.	.	.	.
341	2	69	.	.	.	.	.	.	.	.	0	.	.	.	.	.	.	.	.	.	.	10	1	.	.	.	.	.
342	2	71	.	.	.	.	.	.	.	.	0	.	.	.	.	.	.	.	.	.	.	9	0.9	.	.	.	.	.
343	1	75	.	.	.	.	.	.	.	.	0	.	.	.	.	.	.	.	.	.	.	10	1	.	.	.	.	.
344	1	73	.	.	.	.	.	.	.	.	0	.	.	.	.	.	.	.	.	.	.	10	1	.	.	.	.	.
345	1	70	.	.	.	.	.	.	.	.	0	.	.	.	.	.	.	.	.	.	.	9	0.9	.	.	.	.	.
346	1	75	.	.	.	.	.	.	.	.	0	.	.	.	.	.	.	.	.	.	.	9	0.9	.	.	.	.	.
347	2	73	.	.	.	.	.	.	.	.	0	.	.	.	.	.	.	.	.	.	.	9	0.9	.	.	.	.	.
348	1	74	.	.	.	.	.	.	.	.	0	.	.	.	.	.	.	.	.	.	.	10	1	.	.	.	.	.
349	2	70	.	.	.	.	.	.	.	.	0	.	.	.	.	.	.	.	.	.	.	10	1	.	.	.	.	.
350	2	78	.	.	.	.	.	.	.	.	0	.	.	.	.	.	.	.	.	.	.	9	0.9	.	.	.	.	.
351	2	71	.	.	.	.	.	.	.	.	0	.	.	.	.	.	.	.	.	.	.	8	0.8	.	.	.	.	.
352	1	77	.	.	.	.	.	.	.	.	0	.	.	.	.	.	.	.	.	.	.	10	1	.	.	.	.	.
353	2	72	.	.	.	.	.	.	.	.	0	.	.	.	.	.	.	.	.	.	.	8	0.8	.	.	.	.	.
354	1	68	.	.	.	.	.	.	.	.	0	.	.	.	.	.	.	.	.	.	.	9	0.9	.	.	.	.	.
355	2	72	.	.	.	.	.	.	.	.	0	.	.	.	.	.	.	.	.	.	.	8	0.8	.	.	.	.	.
356	1	76	.	.	.	.	.	.	.	.	7	.	.	.	.	.	.	.	.	.	.	8	0.8	.	.	.	.	.
357	1	73	.	.	.	.	.	.	.	.	0	.	.	.	.	.	.	.	.	.	.	7	0.7	.	.	.	.	.
358	1	67	.	.	.	.	.	.	.	.	0	.	.	.	.	.	.	.	.	.	.	6	0.6	.	.	.	.	.
359	1	69	.	.	.	.	.	.	.	.	0	.	.	.	.	.	.	.	.	.	.	8	0.8	.	.	.	.	.
360	2	76	.	.	.	.	.	.	.	.	0	.	.	.	.	.	.	.	.	.	.	10	1	.	.	.	.	.
361	1	74	.	.	.	.	.	.	.	.	0	.	.	.	.	.	.	.	.	.	.	10	1	.	.	.	.	.
363	2	73	.	.	.	.	.	.	.	.	0	.	.	.	.	.	.	.	.	.	.	8	0.8	.	.	.	.	.
364	2	80	.	.	.	.	.	.	.	.	0	.	.	.	.	.	.	.	.	.	.	10	1	.	.	.	.	.
365	1	75	.	.	.	.	.	.	.	.	0	.	.	.	.	.	.	.	.	.	.	9	0.9	.	.	.	.	.
366	1	73	.	.	.	.	.	.	.	.	0	.	.	.	.	.	.	.	.	.	.	9	0.9	.	.	.	.	.
367	1	79	.	.	.	.	.	.	.	.	0	.	.	.	.	.	.	.	.	.	.	10	1	.	.	.	.	.
368	2	75	.	.	.	.	.	.	.	.	7	.	.	.	.	.	.	.	.	.	.	7	0.7	.	.	.	.	.
371	2	71	.	.	.	.	.	.	.	.	0	.	.	.	.	.	.	.	.	.	.	10	1	.	.	.	.	.
372	2	73	1	1	1	1	1	1	1	1	8	1	1	1	1	1	1	1	0	1	0	8	0.8	1	1	1	1	1
373	2	73	1	1	1	1	1	1	1	1	8	1	1	1	1	1	1	1	1	0	0	8	0.8	1	0	1	1	1
374	2	78	1	1	1	1	1	1	1	1	8	1	1	1	1	1	1	1	1	0	0	8	0.8	1	1	1	1	1
376	2	79	1	1	1	1	1	1	1	1	8	1	1	1	1	1	1	1	1	0	0	8	0.8	1	1	1	1	1
377	2	75	1	1	1	1	1	1	1	1	8	1	1	1	1	1	1	1	0	0	0	7	0.7	1	1	1	0	1
378	1	78	1	1	1	1	1	1	1	1	8	1	1	1	1	1	1	1	1	1	0	9	0.9	1	1	1	1	1
379	1	74	1	1	1	1	1	1	1	1	8	1	1	1	1	1	1	1	0	0	0	7	0.7	1	1	1	1	1
380	2	84	1	1	1	1	1	1	1	1	8	1	1	1	1	1	1	1	1	1	1	10	1	1	1	1	1	1
381	2	70	1	1	1	1	1	1	1	1	8	1	1	1	1	1	1	1	0	0	0	7	0.7	1	1	1	0	0
382	1	69	1	1	1	1	1	1	1	1	8	1	1	1	1	1	1	1	1	1	0	9	0.9	1	1	1	0	1
383	1	78	1	1	1	1	1	1	1	1	8	1	1	1	1	1	1	1	1	1	0	9	0.9	1	1	1	1	1
384	2	76	1	1	1	1	1	1	1	1	8	1	1	1	1	1	1	1	1	1	0	9	0.9	1	1	1	1	1
385	2	79	1	1	1	1	1	1	1	1	8	1	1	1	1	1	1	1	1	1	1	10	1	1	1	1	1	1
386	1	77	1	1	1	1	1	1	1	1	8	1	1	1	1	1	1	1	1	1	1	10	1	1	1	1	1	1
387	2	82	1	1	1	1	1	1	1	1	8	1	1	1	1	1	1	1	1	0	0	8	0.8	1	0	0	1	1
388	1	74	1	1	1	1	1	1	1	1	8	1	1	1	1	1	1	1	1	1	1	10	1	1	1	0	1	1
389	1	79	1	1	1	1	1	1	1	1	8	1	1	1	1	1	1	1	1	1	1	10	1	1	1	1	1	1
390	1	72	1	1	1	1	1	1	1	1	8	1	1	1	1	1	1	1	1	1	0	9	0.9	1	1	1	1	1
391	1	72	1	1	1	1	0	1	1	1	7	1	1	1	1	1	0	1	1	0	0	7	0.7	1	1	1	0	0
392	1	79	1	1	1	1	1	1	1	1	8	1	1	1	1	1	1	1	1	1	1	10	1	1	1	1	1	1
393	1	80	1	1	1	0	1	1	1	1	7	1	1	1	1	1	1	1	1	0	0	8	0.8	1	0	1	1	1
394	1	73	1	1	1	1	1	1	1	1	8	1	1	1	1	1	1	1	1	1	1	10	1	1	1	1	1	1
395	2	80	1	1	1	1	1	1	1	1	8	1	1	1	1	1	1	1	1	0	0	8	0.8	1	1	1	1	1
396	2	77	1	1	1	1	1	1	1	1	8	1	1	1	1	1	1	1	1	1	1	10	1	1	1	1	1	1
397	1	72	1	1	1	1	1	1	1	1	8	1	1	1	1	1	1	1	0	0	0	7	0.7	1	0	1	1	1
399	2	77	1	1	1	1	1	1	1	1	8	1	1	1	1	1	1	1	1	0	0	8	0.8	1	0	0	0	1
400	2	74	1	1	1	1	1	1	1	1	8	1	1	1	1	1	1	1	1	1	0	9	0.9	1	1	1	1	1
401	1	76	1	1	1	1	1	1	1	1	8	1	1	1	1	1	1	1	1	0	0	8	0.8	1	1	1	1	1
402	2	68	1	1	1	1	1	1	1	1	8	1	1	1	1	1	1	1	0	0	0	7	0.7	1	0	1	1	1
403	1	79	1	1	1	1	1	1	1	1	8	1	1	1	1	1	1	1	1	1	0	9	0.9	1	0	1	1	0
404	2	76	1	1	1	1	1	1	1	1	8	1	1	1	1	1	1	1	1	1	0	9	0.9	1	0	1	1	1
405	2	79	1	1	1	1	1	1	1	1	8	1	1	1	1	1	1	1	1	0	0	8	0.8	1	1	1	1	1
406	2	76	1	1	1	1	1	1	1	1	8	1	1	1	1	1	1	1	1	1	1	10	1	1	1	1	1	1
407	1	77	1	1	1	1	1	1	1	1	8	1	1	1	1	1	1	1	1	1	1	10	1	1	1	1	1	1
408	1	77	1	1	1	1	1	1	1	1	8	1	1	1	1	1	1	1	1	1	1	10	1	1	1	1	1	1

comparing_6050	comparing_4090	comparing_5973	comparing_4238	comparing_109180	comparing_403420	comparing_sum	comparing_pomp	counting_tutles	counting_bunnies	counting_sharks	counting_mix	counting_sum	arith_1	arith_2	arith_3	arith_4	arith_5	arith_6	arith_7	arith_8	arith_9	arith_10	arith_11	arith_12	arith_13	arith_14	arith_15
0	1	0	0	0	1	5	0.42	0	1	1	1	3	1	1	0	1	1	0	0	0	0	0	0	0	0	1	0
1	1	0	0	1	1	8	0.67	1	1	1	1	4	1	1	1	1	1	1	1	1	1	1	1	1	0	1	0
1	1	1	0	0	0	8	0.67	1	1	1	1	4	1	1	1	1	1	1	1	1	1	1	1	1	0	1	1
1	1	0	0	0	1	8	0.67	1	1	1	1	4	1	1	1	1	1	1	1	0	1	1	1	1	1	1	0
1	0	1	1	1	1	10	0.83	1	1	1	1	4	1	1	1	1	1	1	1	1	0	0	1	1	1	1	1
0	1	0	0	0	1	7	0.58	1	1	1	1	4	1	1	1	1	1	1	1	1	1	1	1	1	1	1	1
1	1	1	0	0	0	8	0.67	1	1	1	0	3	1	1	1	1	1	1	1	1	1	1	1	1	1	1	1
0	0	1	0	0	1	6	0.5	1	1	1	1	4	0	1	0	1	0	1	0	0	0	1	0	0	0	0	0
1	1	1	0	1	1	9	0.75	0	1	1	1	3	0	0	0	0	0	0	0	0	0	0	0	0	0	0	0
0	0	0	0	0	1	4	0.33	1	1	1	1	4	1	1	1	1	1	1	1	1	1	1	1	1	1	1	1
1	0	0	1	1	0	7	0.58	0	1	1	1	3	1	1	1	1	0	0	1	1	0	0	1	0	0	1	1
1	1	0	1	0	1	8	0.67	1	1	1	1	4	1	1	1	1	1	1	1	1	1	1	1	1	1	1	1
1	1	0	0	1	0	7	0.58	1	1	1	1	4	1	1	1	1	1	1	1	1	1	1	1	1	1	1	1
1	1	0	0	0	0	7	0.58	1	1	1	1	4	1	1	1	1	1	1	1	1	1	1	1	1	1	1	1
1	1	1	0	0	1	9	0.75	1	1	1	1	4	0	0	0	0	1	0	0	1	0	0	0	0	0	0	0
1	1	0	0	0	0	7	0.58	1	1	1	1	4	1	1	1	1	1	1	1	1	1	1	1	1	1	1	0
1	1	1	0	0	0	8	0.67	1	1	1	1	4	0	0	1	1	1	1	1	1	1	1	1	0	0	0	0
1	1	0	0	0	1	5	0.42	1	1	1	1	4	0	0	0	1	0	0	0	0	0	0	0	0	0	0	1
1	1	0	0	0	1	5	0.42	1	1	1	1	4	0	1	1	1	1	1	1	0	1	0	1	0	0	0	0
0	0	0	1	0	0	3	0.25	1	1	1	1	4	0	0	0	1	0	0	1	0	0	0	0	0	1	0	0
1	1	0	0	0	1	8	0.67	1	1	1	1	4	1	1	1	1	1	0	1	1	1	0	1	1	0	1	0
1	1	0	0	0	0	7	0.58	1	1	1	1	4	1	1	1	1	1	1	1	1	1	1	1	1	1	1	1
1	1	1	0	0	0	8	0.67	1	1	1	1	4	1	1	1	1	1	0	1	0	1	1	1	0	0	1	0
0	1	1	0	1	1	8	0.67	0	1	1	1	3	0	0	0	1	0	0	0	0	1	0	0	0	0	0	0
1	1	1	1	0	1	10	0.83	1	1	1	1	4	1	1	1	1	1	1	1	1	1	1	1	1	1	0	1
1	1	0	1	0	0	8	0.67	1	1	1	1	4	1	1	1	1	1	1	1	1	1	1	1	1	1	1	1
1	1	1	0	0	1	9	0.75	1	1	1	1	4	1	1	1	1	1	1	1	1	1	1	1	1	1	1	1
1	1	1	1	1	1	11	0.92	1	1	1	1	4	1	1	1	1	1	1	1	1	1	1	1	1	1	1	1
1	1	1	1	0	1	10	0.83	1	1	1	1	4	1	1	0	1	1	1	1	1	1	1	1	1	1	1	1
1	1	1	0	1	0	9	0.75	1	1	0	1	3	1	1	1	1	1	1	1	1	1	1	0	1	1	1	1
1	1	0	0	1	1	9	0.75	1	1	1	1	4	1	1	0	1	1	1	1	1	1	1	1	1	1	1	1
1	1	1	1	0	0	9	0.75	1	1	1	1	4	1	1	1	1	1	1	1	1	0	0	1	1	1	1	1
1	1	1	1	1	1	11	0.92	1	1	1	1	4	1	1	1	1	1	1	1	1	1	1	1	1	1	1	1
1	1	0	0	1	1	8	0.67	1	1	1	1	4	1	1	1	1	1	1	1	1	1	0	1	0	1	1	0
0	0	0	0	0	0	5	0.42	0	1	1	1	3	0	1	1	1	1	0	1	0	1	0	1	0	0	1	0
0	1	0	0	0	0	6	0.5	1	1	1	1	4	1	1	1	1	1	1	1	1	1	1	1	1	1	1	1
1	1	1	0	1	1	9	0.75	1	1	1	1	4	1	1	1	1	1	1	1	1	1	1	1	1	1	1	0
1	1	1	1	1	1	11	0.92	1	1	1	1	4	1	1	1	1	1	1	1	1	1	1	1	1	0	1	1
1	1	0	0	0	0	7	0.58	0	1	1	1	3	1	1	0	1	1	0	1	0	1	1	1	0	0	1	1
1	1	1	0	0	0	6	0.5	1	1	1	1	4	0	0	0	0	0	0	0	0	0	0	0	0	0	0	1
1	1	1	1	0	0	9	0.75	1	1	1	1	4	1	1	0	1	0	1	1	1	1	1	1	1	1	1	1
1	1	1	0	1	0	9	0.75	1	1	1	1	4	0	1	1	1	1	1	1	1	1	1	1	1	0	1	1
1	1	1	1	1	1	11	0.92	1	1	1	1	4	1	1	0	1	1	1	1	1	1	1	1	1	1	1	1
1	1	0	0	1	1	9	0.75	1	1	1	1	4	1	1	1	1	1	0	0	0	0	1	1	1	1	1	1
0	0	0	0	0	0	5	0.42	0	1	1	1	3	1	1	1	1	1	1	1	1	1	1	1	1	1	1	1
1	0	1	0	0	1	8	0.67	1	1	1	1	4	0	0	0	0	0	1	1	1	1	1	1	0	0	0	0
0	1	0	0	0	1	7	0.58	1	1	1	1	4	1	1	0	1	1	1	1	1	1	1	1	1	0	1	0
0	1	0	0	0	1	6	0.5	1	1	1	0	3	1	1	1	1	1	1	1	1	1	1	1	1	1	1	1
0	1	1	0	0	0	4	0.33	1	0	1	1	3	0	0	0	0	0	0	1	0	0	0	0	0	0	0	0
0	0	0	1	0	0	2	0.17	1	1	1	1	4	1	0	0	0	0	0	0	0	0	0	0	0	0	0	0
0	0	0	0	0	0	1	0.08	1	1	1	1	4	0	0	0	1	0	0	1	0	0	0	0	0	0	0	0
0	0	0	0	0	0	5	0.42	1	1	1	1	4	0	0	0	0	0	0	0	0	0	0	0	0	0	0	0
1	1	0	1	1	0	7	0.58	1	1	1	1	4	1	1	1	1	1	1	1	1	1	1	1	1	1	1	1
1	1	0	0	1	0	8	0.67	1	1	1	1	4	1	1	1	1	0	1	1	0	1	1	1	1	1	1	1
1	1	1	0	1	1	9	0.75	1	1	1	1	4	1	1	0	1	1	1	1	1	1	1	1	1	1	1	1
1	1	0	0	0	0	7	0.58	1	1	1	1	4	1	1	1	1	1	1	1	1	1	1	1	1	1	1	1
1	0	0	1	1	1	8	0.67	0	1	1	0	2	1	1	0	1	0	0	1	0	1	0	1	0	0	1	0
1	1	1	0	1	1	9	0.75	1	1	1	1	4	0	0	0	1	0	1	1	0	0	0	1	0	0	1	0
1	1	0	0	0	0	7	0.58	1	1	1	1	4	0	1	1	1	1	0	1	1	0	1	1	1	1	1	1
1	1	0	0	0	0	7	0.58	1	1	1	1	4	1	1	1	1	1	1	1	1	1	1	1	0	1	1	1
1	1	1	1	1	1	11	0.92	1	1	1	1	4	1	1	1	1	1	1	1	1	1	1	1	1	1	1	1
1	1	1	1	1	1	11	0.92	1	1	1	1	4	1	0	0	0	0	0	0	0	0	0	0	1	0	1	0
1	1	1	1	0	0	9	0.75	1	1	1	1	4	1	0	1	1	1	1	1	0	1	1	0	1	1	0	1
1	1	1	1	0	0	9	0.75	1	1	1	1	4	1	1	1	1	1	1	1	1	1	1	1	1	1	1	1
1	1	1	1	1	1	11	0.92	1	1	1	1	4	1	1	1	1	1	1	1	1	1	1	1	1	1	1	1
1	1	1	1	1	1	11	0.92	1	1	1	1	4	1	1	1	1	1	1	1	1	1	1	1	1	1	1	1
1	1	0	0	0	0	7	0.58	1	1	1	1	4	1	1	0	1	1	1	1	1	1	1	1	1	1	1	1
0	1	0	0	0	0	5	0.42	1	1	1	1	4	1	0	1	1	1	1	1	1	1	1	0	1	1	0	1

1	1	1	0	1	1	10	0.83	1	1	1	1	4	1	0	1	1	1	1	1	1	1	1	0	1	0	0	1
1	1	1	1	1	1	11	0.92	1	1	1	1	4	1	1	1	1	1	1	1	1	1	1	1	1	1	1	1
1	0	0	0	0	0	4	0.33	1	1	1	1	4	1	1	1	0	0	0	0	0	0	0	1	0	0	0	0
1	1	0	0	0	0	5	0.42	1	1	1	1	4	1	1	1	1	1	1	1	1	1	1	1	1	0	0	0
0	1	1	0	0	0	7	0.58	1	1	1	1	4	1	0	0	1	0	0	0	0	0	0	0	1	0	0	0
1	1	1	1	1	1	11	0.92	1	1	1	1	4	1	1	1	1	1	1	1	1	1	1	1	1	1	1	1
1	1	1	0	1	1	10	0.83	1	1	1	1	4	1	0	1	1	1	1	1	1	1	1	0	1	1	0	1
1	1	1	0	0	0	8	0.67	1	1	1	1	4	1	0	1	1	1	1	1	1	0	0	0	1	1	0	1
1	1	0	0	1	1	9	0.75	1	1	1	1	4	1	1	1	1	1	1	1	1	1	1	1	1	1	1	1
1	1	1	0	0	1	9	0.75	1	1	1	1	4	1	0	1	1	1	1	1	1	1	1	0	1	0	0	1
1	1	1	1	1	1	11	0.92	0	1	1	1	3	1	1	1	1	1	1	1	1	1	1	1	0	1	1	1
1	1	0	0	1	1	9	0.75	1	1	1	1	4	1	0	0	0	0	0	0	0	0	0	0	0	0	0	0
1	0	1	0	1	0	8	0.67	1	0	1	1	3	1	0	1	0	1	0	1	0	1	1	0	0	0	0	1
1	1	0	0	1	1	9	0.75	1	1	1	1	4	1	1	1	1	1	0	1	0	1	0	1	1	0	1	1
0	0	0	0	0	0	5	0.42	1	1	1	1	4	0	0	0	0	0	1	0	0	0	0	1	0	0	0	0
1	1	0	0	1	0	6	0.5	1	1	1	1	4	0	1	1	1	1	0	0	1	0	0	0	0	0	0	0
1	1	0	1	1	0	8	0.67	1	1	1	1	4	1	1	1	1	1	1	0	0	0	0	0	0	0	0	0
1	1	0	0	0	0	7	0.58	1	1	1	1	4	1	1	1	1	1	1	1	1	1	1	1	1	1	1	1
1	1	0	0	1	0	8	0.67	1	1	1	0	3	0	0	0	0	0	1	0	0	0	0	0	0	0	0	0
1	1	0	0	0	0	7	0.58	1	1	1	1	4	1	0	0	0	0	0	0	0	0	0	1	1	0	0	0
1	1	0	0	0	0	7	0.58	1	1	1	1	4	1	0	1	1	1	1	1	1	1	1	1	1	0	1	0
1	1	1	1	1	1	11	0.92	1	1	1	1	4	1	1	1	1	1	1	1	1	1	1	1	1	1	1	1
1	1	1	1	1	1	11	0.92	1	1	1	1	4	0	0	0	0	0	0	1	0	0	0	0	0	0	0	0
0	0	1	0	1	0	4	0.33	1	1	1	1	4	1	1	1	1	1	1	1	1	1	1	1	0	1	1	0
0	0	1	1	1	1	7	0.58	1	1	1	0	3	1	1	1	1	1	1	1	1	1	1	1	1	1	1	0
1	1	1	0	0	0	8	0.67	1	1	1	1	4	1	0	0	0	0	0	0	0	0	0	0	1	0	0	0
1	0	1	0	1	1	8	0.67	1	0	0	1	2	1	0	1	0	1	0	0	0	0	0	0	0	0	0	1
1	1	1	1	1	0	8	0.67	1	0	0	1	2	0	0	0	0	0	0	0	0	0	0	1	0	0	0	1
1	1	1	1	1	1	11	0.92	1	1	1	1	4	1	1	1	1	1	1	1	1	1	1	1	1	1	1	1
0	1	1	0	0	1	7	0.58	1	1	1	1	4	1	1	1	1	1	1	1	1	1	0	1	1	1	1	1
1	1	1	1	0	0	9	0.75	1	1	1	1	4	1	1	1	1	1	1	1	1	1	1	1	1	1	1	1
1	1	1	1	1	1	11	0.92	1	1	1	1	4	1	1	1	1	1	1	1	1	1	1	1	1	1	0	1
1	1	1	1	1	1	11	0.92	1	1	1	1	4	1	1	1	1	1	1	1	1	1	1	1	1	1	1	0
1	1	1	1	0	1	10	0.83	1	1	1	1	4	1	1	1	1	1	1	1	1	1	1	1	1	1	1	1
1	1	1	1	0	1	10	0.83	1	1	1	1	4	1	1	1	1	1	1	1	1	1	1	1	1	1	1	1
1	1	0	0	0	0	7	0.58	1	1	1	1	4	1	1	1	1	1	1	1	1	1	1	1	1	0	1	0
1	1	1	1	1	1	11	0.92	1	1	1	1	4	1	0	1	1	1	1	1	1	1	1	0	1	1	0	0
1	1	1	1	1	0	10	0.83	1	1	1	1	4	1	0	1	1	1	1	1	1	1	1	1	1	1	1	1
1	1	1	1	1	0	10	0.83	1	1	1	1	4	1	1	1	1	1	1	1	1	1	1	1	1	1	1	1
1	1	1	1	0	0	8	0.67	1	1	1	1	4	0	0	0	0	0	0	0	1	0	0	0	0	0	0	0
0	0	0	0	0	0	3	0.25	1	1	1	0	3	1	1	1	1	1	1	0	0	0	0	0	0	0	0	0
1	1	1	0	0	0	8	0.67	1	1	1	1	4	1	1	0	0	0	0	0	0	0	0	0	0	0	0	0
1	1	0	0	0	0	7	0.58	1	1	1	1	4	1	1	0	0	0	0	0	0	0	0	0	0	0	0	0
1	1	1	0	1	0	8	0.67	0	0	1	1	2	1	1	1	1	1	1	1	1	1	1	1	1	1	1	1
1	1	1	1	0	0	9	0.75	1	1	1	1	4	0	1	1	1	1	1	1	0	1	1	1	1	1	1	0
1	1	0	1	0	1	9	0.75	1	1	1	1	4	1	1	1	1	1	1	1	1	1	1	1	1	1	1	1
1	1	1	0	1	0	9	0.75	1	1	1	1	4	1	1	1	1	1	1	1	1	1	1	1	1	1	1	1
1	1	1	1	1	1	11	0.92	1	1	1	1	4	1	1	0	1	1	1	1	1	1	1	1	1	0	1	1
0	0	0	0	0	0	4	0.33	1	1	1	0	3	1	0	0	0	0	0	0	0	0	0	0	0	0	0	0
1	1	0	0	0	0	7	0.58	1	1	1	1	4	1	1	1	1	1	1	1	1	1	1	1	1	1	1	1
1	1	0	1	0	0	8	0.67	1	1	1	1	4	1	0	0	0	1	1	0	1	1	0	0	0	0	0	0
1	1	1	1	1	1	11	0.92	1	1	1	1	4	0	0	0	1	0	0	1	0	0	0	0	0	0	0	0
1	1	0	0	0	0	7	0.58	1	1	1	1	4	1	1	1	1	1	1	1	1	1	1	1	1	1	1	1
1	1	0	0	0	0	7	0.58	1	1	0	1	3	0	0	0	0	0	0	0	0	0	0	0	0	0	0	0
1	1	0	0	0	1	8	0.67	1	1	1	1	4	1	1	1	1	1	1	1	1	1	0	1	0	0	0	0
.	.	.	.	.	.	10	0.83	1	1	1	1	4	.	.	.	.	.	.	.	.	.	.	.	.	.	.	.
.	.	.	.	.	.	10	0.83	1	1	1	1	4	.	.	.	.	.	.	.	.	.	.	.	.	.	.	.
.	.	.	.	.	.	0	0	1	1	1	1	4	.	.	.	.	.	.	.	.	.	.	.	.	.	.	.
.	.	.	.	.	.	5	0.42	1	1	1	0	3	.	.	.	.	.	.	.	.	.	.	.	.	.	.	.
.	.	.	.	.	.	3	0.25	1	1	1	1	4	.	.	.	.	.	.	.	.	.	.	.	.	.	.	.
.	.	.	.	.	.	10	0.83	1	1	1	1	4	.	.	.	.	.	.	.	.	.	.	.	.	.	.	.
.	.	.	.	.	.	4	0.33	0	0	0	1	1	.	.	.	.	.	.	.	.	.	.	.	.	.	.	.
.	.	.	.	.	.	9	0.75	1	1	1	1	4	.	.	.	.	.	.	.	.	.	.	.	.	.	.	.
.	.	.	.	.	.	6	0.5	1	1	1	1	4	.	.	.	.	.	.	.	.	.	.	.	.	.	.	.
.	.	.	.	.	.	8	0.67	1	1	1	1	4	.	.	.	.	.	.	.	.	.	.	.	.	.	.	.
.	.	.	.	.	.	8	0.67	1	1	1	0	3	.	.	.	.	.	.	.	.	.	.	.	.	.	.	.
.	.	.	.	.	.	10	0.83	1	1	1	1	4	.	.	.	.	.	.	.	.	.	.	.	.	.	.	.
.	.	.	.	.	.	5	0.42	1	1	1	1	4	.	.	.	.	.	.	.	.	.	.	.	.	.	.	.
.	.	.	.	.	.	10	0.83	1	1	1	1	4	.	.	.	.	.	.	.	.	.	.	.	.	.	.	.

.	.	.	.	.	.	11	0.92	1	1	1	1	4	.	.	.	.	.	.	.	.	.	.	.	.	.	.	.
.	.	.	.	.	.	8	0.67	1	1	1	1	4	.	.	.	.	.	.	.	.	.	.	.	.	.	.	.
.	.	.	.	.	.	8	0.67	1	0	1	0	2	.	.	.	.	.	.	.	.	.	.	.	.	.	.	.
.	.	.	.	.	.	12	1	1	1	1	1	4	.	.	.	.	.	.	.	.	.	.	.	.	.	.	.
.	.	.	.	.	.	0	0	1	1	1	1	4	.	.	.	.	.	.	.	.	.	.	.	.	.	.	.
.	.	.	.	.	.	3	0.25	0	1	1	1	3	.	.	.	.	.	.	.	.	.	.	.	.	.	.	.
.	.	.	.	.	.	10	0.83	1	1	1	1	4	.	.	.	.	.	.	.	.	.	.	.	.	.	.	.
.	.	.	.	.	.	2	0.17	1	1	1	1	4	.	.	.	.	.	.	.	.	.	.	.	.	.	.	.
.	.	.	.	.	.	7	0.58	1	1	1	1	4	.	.	.	.	.	.	.	.	.	.	.	.	.	.	.
.	.	.	.	.	.	7	0.58	1	1	1	1	4	.	.	.	.	.	.	.	.	.	.	.	.	.	.	.
.	.	.	.	.	.	9	0.75	1	1	1	1	4	.	.	.	.	.	.	.	.	.	.	.	.	.	.	.
.	.	.	.	.	.	8	0.67	0	1	1	1	3	.	.	.	.	.	.	.	.	.	.	.	.	.	.	.
.	.	.	.	.	.	5	0.42	1	1	1	1	4	.	.	.	.	.	.	.	.	.	.	.	.	.	.	.
.	.	.	.	.	.	12	1	1	1	1	1	4	.	.	.	.	.	.	.	.	.	.	.	.	.	.	.
.	.	.	.	.	.	8	0.67	1	1	1	1	4	.	.	.	.	.	.	.	.	.	.	.	.	.	.	.
.	.	.	.	.	.	9	0.75	1	0	0	1	2	.	.	.	.	.	.	.	.	.	.	.	.	.	.	.
.	.	.	.	.	.	10	0.83	1	1	1	1	4	.	.	.	.	.	.	.	.	.	.	.	.	.	.	.
.	.	.	.	.	.	11	0.92	0	1	1	1	3	.	.	.	.	.	.	.	.	.	.	.	.	.	.	.
.	.	.	.	.	.	9	0.75	1	1	1	1	4	.	.	.	.	.	.	.	.	.	.	.	.	.	.	.
.	.	.	.	.	.	9	0.75	0	1	1	1	3	.	.	.	.	.	.	.	.	.	.	.	.	.	.	.
.	.	.	.	.	.	8	0.67	0	1	1	1	3	.	.	.	.	.	.	.	.	.	.	.	.	.	.	.
.	.	.	.	.	.	8	0.67	1	1	1	1	4	.	.	.	.	.	.	.	.	.	.	.	.	.	.	.
.	.	.	.	.	.	6	0.5	1	1	1	1	4	.	.	.	.	.	.	.	.	.	.	.	.	.	.	.
.	.	.	.	.	.	11	0.92	1	1	1	1	4	.	.	.	.	.	.	.	.	.	.	.	.	.	.	.
.	.	.	.	.	.	0	0	1	1	1	1	4	.	.	.	.	.	.	.	.	.	.	.	.	.	.	.
.	.	.	.	.	.	10	0.83	1	1	1	1	4	.	.	.	.	.	.	.	.	.	.	.	.	.	.	.
.	.	.	.	.	.	2	0.17	0	0	1	1	2	.	.	.	.	.	.	.	.	.	.	.	.	.	.	.
.	.	.	.	.	.	0	0	1	1	1	1	4	.	.	.	.	.	.	.	.	.	.	.	.	.	.	.
.	.	.	.	.	.	9	0.75	1	1	1	1	4	.	.	.	.	.	.	.	.	.	.	.	.	.	.	.
.	.	.	.	.	.	8	0.67	1	1	1	1	4	.	.	.	.	.	.	.	.	.	.	.	.	.	.	.
.	.	.	.	.	.	9	0.75	1	1	1	1	4	.	.	.	.	.	.	.	.	.	.	.	.	.	.	.
.	.	.	.	.	.	8	0.67	1	1	1	1	4	.	.	.	.	.	.	.	.	.	.	.	.	.	.	.
.	.	.	.	.	.	10	0.83	1	1	1	1	4	.	.	.	.	.	.	.	.	.	.	.	.	.	.	.
.	.	.	.	.	.	1	0.08	1	1	1	1	4	.	.	.	.	.	.	.	.	.	.	.	.	.	.	.
.	.	.	.	.	.	8	0.67	0	1	1	0	2	.	.	.	.	.	.	.	.	.	.	.	.	.	.	.
.	.	.	.	.	.	8	0.67	1	1	1	1	4	.	.	.	.	.	.	.	.	.	.	.	.	.	.	.
.	.	.	.	.	.	2	0.17	1	1	1	1	4	.	.	.	.	.	.	.	.	.	.	.	.	.	.	.
.	.	.	.	.	.	8	0.67	1	1	1	1	4	.	.	.	.	.	.	.	.	.	.	.	.	.	.	.
.	.	.	.	.	.	12	1	1	1	1	1	4	.	.	.	.	.	.	.	.	.	.	.	.	.	.	.
.	.	.	.	.	.	12	1	1	1	1	1	4	.	.	.	.	.	.	.	.	.	.	.	.	.	.	.
.	.	.	.	.	.	10	0.83	1	1	1	1	4	.	.	.	.	.	.	.	.	.	.	.	.	.	.	.
.	.	.	.	.	.	10	0.83	0	1	1	0	2	.	.	.	.	.	.	.	.	.	.	.	.	.	.	.
.	.	.	.	.	.	8	0.67	1	1	1	1	4	.	.	.	.	.	.	.	.	.	.	.	.	.	.	.
.	.	.	.	.	.	9	0.75	0	1	1	1	3	.	.	.	.	.	.	.	.	.	.	.	.	.	.	.
.	.	.	.	.	.	9	0.75	1	1	1	1	4	.	.	.	.	.	.	.	.	.	.	.	.	.	.	.
.	.	.	.	.	.	8	0.67	1	1	1	1	4	.	.	.	.	.	.	.	.	.	.	.	.	.	.	.
.	.	.	.	.	.	9	0.75	1	1	1	1	4	.	.	.	.	.	.	.	.	.	.	.	.	.	.	.
.	.	.	.	.	.	7	0.58	0	0	0	1	1	.	.	.	.	.	.	.	.	.	.	.	.	.	.	.
.	.	.	.	.	.	9	0.75	1	1	1	1	4	.	.	.	.	.	.	.	.	.	.	.	.	.	.	.
.	.	.	.	.	.	12	1	1	1	1	1	4	.	.	.	.	.	.	.	.	.	.	.	.	.	.	.
.	.	.	.	.	.	8	0.67	1	1	1	1	4	.	.	.	.	.	.	.	.	.	.	.	.	.	.	.
.	.	.	.	.	.	9	0.75	1	1	1	1	4	.	.	.	.	.	.	.	.	.	.	.	.	.	.	.
.	.	.	.	.	.	9	0.75	1	1	1	1	4	.	.	.	.	.	.	.	.	.	.	.	.	.	.	.
.	.	.	.	.	.	10	0.83	0	1	0	1	2	.	.	.	.	.	.	.	.	.	.	.	.	.	.	.
.	.	.	.	.	.	7	0.58	1	1	1	1	4	.	.	.	.	.	.	.	.	.	.	.	.	.	.	.
.	.	.	.	.	.	0	0	1	1	1	1	4	.	.	.	.	.	.	.	.	.	.	.	.	.	.	.
.	.	.	.	.	.	8	0.67	0	1	1	1	3	.	.	.	.	.	.	.	.	.	.	.	.	.	.	.
.	.	.	.	.	.	9	0.75	1	1	1	0	3	.	.	.	.	.	.	.	.	.	.	.	.	.	.	.
.	.	.	.	.	.	9	0.75	0	1	1	1	3	.	.	.	.	.	.	.	.	.	.	.	.	.	.	.
.	.	.	.	.	.	8	0.67	1	1	1	1	4	.	.	.	.	.	.	.	.	.	.	.	.	.	.	.
.	.	.	.	.	.	9	0.75	1	1	1	1	4	.	.	.	.	.	.	.	.	.	.	.	.	.	.	.
.	.	.	.	.	.	9	0.75	1	1	1	1	4	.	.	.	.	.	.	.	.	.	.	.	.	.	.	.
.	.	.	.	.	.	12	1	1	1	1	0	3	.	.	.	.	.	.	.	.	.	.	.	.	.	.	.
.	.	.	.	.	.	9	0.75	1	1	1	1	4	.	.	.	.	.	.	.	.	.	.	.	.	.	.	.
.	.	.	.	.	.	12	1	1	1	1	1	4	.	.	.	.	.	.	.	.	.	.	.	.	.	.	.
.	.	.	.	.	.	10	0.83	1	1	1	1	4	.	.	.	.	.	.	.	.	.	.	.	.	.	.	.
.	.	.	.	.	.	10	0.83	1	1	1	1	4	.	.	.	.	.	.	.	.	.	.	.	.	.	.	.
.	.	.	.	.	.	11	0.92	1	1	1	1	4	.	.	.	.	.	.	.	.	.	.	.	.	.	.	.
.	.	.	.	.	.	8	0.67	1	1	1	1	4	.	.	.	.	.	.	.	.	.	.	.	.	.	.	.
.	.	.	.	.	.	8	0.67	1	1	1	1	4	.	.	.	.	.	.	.	.	.	.	.	.	.	.	.

.	.	.	.	.	.	7	0.58	1	1	1	1	4	.	.	.	.	.	.	.	.	.	.	.	.	.	.	.
.	.	.	.	.	.	7	0.58	1	1	1	1	4	.	.	.	.	.	.	.	.	.	.	.	.	.	.	.
.	.	.	.	.	.	12	1	1	1	1	1	4	.	.	.	.	.	.	.	.	.	.	.	.	.	.	.
.	.	.	.	.	.	9	0.75	1	1	1	1	4	.	.	.	.	.	.	.	.	.	.	.	.	.	.	.
.	.	.	.	.	.	6	0.5	1	1	1	1	4	.	.	.	.	.	.	.	.	.	.	.	.	.	.	.
.	.	.	.	.	.	9	0.75	1	1	1	1	4	.	.	.	.	.	.	.	.	.	.	.	.	.	.	.
.	.	.	.	.	.	9	0.75	1	1	1	1	4	.	.	.	.	.	.	.	.	.	.	.	.	.	.	.
.	.	.	.	.	.	9	0.75	1	1	1	1	4	.	.	.	.	.	.	.	.	.	.	.	.	.	.	.
.	.	.	.	.	.	8	0.67	1	1	1	1	4	.	.	.	.	.	.	.	.	.	.	.	.	.	.	.
.	.	.	.	.	.	9	0.75	1	1	1	1	4	.	.	.	.	.	.	.	.	.	.	.	.	.	.	.
.	.	.	.	.	.	10	0.83	1	1	1	1	4	.	.	.	.	.	.	.	.	.	.	.	.	.	.	.
.	.	.	.	.	.	12	1	1	1	1	1	4	.	.	.	.	.	.	.	.	.	.	.	.	.	.	.
.	.	.	.	.	.	9	0.75	1	1	1	1	4	.	.	.	.	.	.	.	.	.	.	.	.	.	.	.
.	.	.	.	.	.	9	0.75	1	1	1	1	4	.	.	.	.	.	.	.	.	.	.	.	.	.	.	.
.	.	.	.	.	.	10	0.83	1	1	1	1	4	.	.	.	.	.	.	.	.	.	.	.	.	.	.	.
.	.	.	.	.	.	12	1	1	1	1	1	4	.	.	.	.	.	.	.	.	.	.	.	.	.	.	.
.	.	.	.	.	.	10	0.83	1	1	1	1	4	.	.	.	.	.	.	.	.	.	.	.	.	.	.	.
.	.	.	.	.	.	12	1	1	1	1	1	4	.	.	.	.	.	.	.	.	.	.	.	.	.	.	.
.	.	.	.	.	.	12	1	1	1	1	1	4	.	.	.	.	.	.	.	.	.	.	.	.	.	.	.
.	.	.	.	.	.	10	0.83	1	1	1	1	4	.	.	.	.	.	.	.	.	.	.	.	.	.	.	.
.	.	.	.	.	.	9	0.75	1	1	1	1	4	.	.	.	.	.	.	.	.	.	.	.	.	.	.	.
.	.	.	.	.	.	10	0.83	1	1	1	1	4	.	.	.	.	.	.	.	.	.	.	.	.	.	.	.
.	.	.	.	.	.	8	0.67	1	1	1	1	4	.	.	.	.	.	.	.	.	.	.	.	.	.	.	.
.	.	.	.	.	.	8	0.67	1	1	1	1	4	.	.	.	.	.	.	.	.	.	.	.	.	.	.	.
.	.	.	.	.	.	9	0.75	1	1	1	1	4	.	.	.	.	.	.	.	.	.	.	.	.	.	.	.
.	.	.	.	.	.	12	1	1	1	1	1	4	.	.	.	.	.	.	.	.	.	.	.	.	.	.	.
.	.	.	.	.	.	12	1	1	1	1	1	4	.	.	.	.	.	.	.	.	.	.	.	.	.	.	.
.	.	.	.	.	.	11	0.92	1	1	1	1	4	.	.	.	.	.	.	.	.	.	.	.	.	.	.	.
.	.	.	.	.	.	10	0.83	1	1	1	1	4	.	.	.	.	.	.	.	.	.	.	.	.	.	.	.
.	.	.	.	.	.	10	0.83	1	1	1	1	4	.	.	.	.	.	.	.	.	.	.	.	.	.	.	.
.	.	.	.	.	.	7	0.58	0	1	1	1	3	.	.	.	.	.	.	.	.	.	.	.	.	.	.	.
.	.	.	.	.	.	7	0.58	1	1	1	1	4	.	.	.	.	.	.	.	.	.	.	.	.	.	.	.
.	.	.	.	.	.	5	0.42	1	1	1	1	4	.	.	.	.	.	.	.	.	.	.	.	.	.	.	.
.	.	.	.	.	.	10	0.83	0	1	1	0	2	.	.	.	.	.	.	.	.	.	.	.	.	.	.	.
.	.	.	.	.	.	8	0.67	1	1	1	1	4	.	.	.	.	.	.	.	.	.	.	.	.	.	.	.
.	.	.	.	.	.	11	0.92	1	1	1	1	4	.	.	.	.	.	.	.	.	.	.	.	.	.	.	.
.	.	.	.	.	.	6	0.5	1	1	1	1	4	.	.	.	.	.	.	.	.	.	.	.	.	.	.	.
.	.	.	.	.	.	7	0.58	1	1	1	1	4	.	.	.	.	.	.	.	.	.	.	.	.	.	.	.
.	.	.	.	.	.	8	0.67	0	1	0	0	1	.	.	.	.	.	.	.	.	.	.	.	.	.	.	.
.	.	.	.	.	.	6	0.5	1	1	1	1	4	.	.	.	.	.	.	.	.	.	.	.	.	.	.	.
.	.	.	.	.	.	8	0.67	0	1	1	1	3	.	.	.	.	.	.	.	.	.	.	.	.	.	.	.
.	.	.	.	.	.	9	0.75	0	1	1	1	3	.	.	.	.	.	.	.	.	.	.	.	.	.	.	.
.	.	.	.	.	.	6	0.5	1	1	1	1	4	.	.	.	.	.	.	.	.	.	.	.	.	.	.	.
.	.	.	.	.	.	9	0.75	1	1	1	1	4	.	.	.	.	.	.	.	.	.	.	.	.	.	.	.
.	.	.	.	.	.	9	0.75	0	1	1	1	3	.	.	.	.	.	.	.	.	.	.	.	.	.	.	.
.	.	.	.	.	.	10	0.83	1	1	1	1	4	.	.	.	.	.	.	.	.	.	.	.	.	.	.	.
.	.	.	.	.	.	7	0.58	1	1	1	1	4	.	.	.	.	.	.	.	.	.	.	.	.	.	.	.
.	.	.	.	.	.	10	0.83	1	1	1	1	4	.	.	.	.	.	.	.	.	.	.	.	.	.	.	.
.	.	.	.	.	.	11	0.92	1	1	1	1	4	.	.	.	.	.	.	.	.	.	.	.	.	.	.	.
.	.	.	.	.	.	9	0.75	1	1	1	1	4	.	.	.	.	.	.	.	.	.	.	.	.	.	.	.
.	.	.	.	.	.	8	0.67	0	1	1	1	3	.	.	.	.	.	.	.	.	.	.	.	.	.	.	.
.	.	.	.	.	.	9	0.75	1	1	1	1	4	.	.	.	.	.	.	.	.	.	.	.	.	.	.	.
.	.	.	.	.	.	9	0.75	1	1	1	1	4	.	.	.	.	.	.	.	.	.	.	.	.	.	.	.
.	.	.	.	.	.	9	0.75	1	1	1	1	4	.	.	.	.	.	.	.	.	.	.	.	.	.	.	.
.	.	.	.	.	.	10	0.83	1	1	1	1	4	.	.	.	.	.	.	.	.	.	.	.	.	.	.	.
.	.	.	.	.	.	8	0.67	1	1	1	1	4	.	.	.	.	.	.	.	.	.	.	.	.	.	.	.
.	.	.	.	.	.	9	0.75	1	1	1	1	4	.	.	.	.	.	.	.	.	.	.	.	.	.	.	.
.	.	.	.	.	.	6	0.5	1	1	1	1	4	.	.	.	.	.	.	.	.	.	.	.	.	.	.	.
.	.	.	.	.	.	9	0.75	1	1	1	1	4	.	.	.	.	.	.	.	.	.	.	.	.	.	.	.
.	.	.	.	.	.	8	0.67	0	1	1	1	3	.	.	.	.	.	.	.	.	.	.	.	.	.	.	.
.	.	.	.	.	.	9	0.75	1	1	1	1	4	.	.	.	.	.	.	.	.	.	.	.	.	.	.	.
.	.	.	.	.	.	10	0.83	1	1	1	1	4	.	.	.	.	.	.	.	.	.	.	.	.	.	.	.
.	.	.	.	.	.	12	1	1	0	0	0	1	.	.	.	.	.	.	.	.	.	.	.	.	.	.	.
.	.	.	.	.	.	10	0.83	1	1	1	1	4	.	.	.	.	.	.	.	.	.	.	.	.	.	.	.
.	.	.	.	.	.	12	1	1	1	1	1	4	.	.	.	.	.	.	.	.	.	.	.	.	.	.	.
.	.	.	.	.	.	9	0.75	1	0	1	1	3	.	.	.	.	.	.	.	.	.	.	.	.	.	.	.
.	.	.	.	.	.	9	0.75	1	1	1	1	4	.	.	.	.	.	.	.	.	.	.	.	.	.	.	.
.	.	.	.	.	.	8	0.67	1	1	1	1	4	.	.	.	.	.	.	.	.	.	.	.	.	.	.	.
.	.	.	.	.	.	11	0.92	1	1	1	1	4	.	.	.	.	.	.	.	.	.	.	.	.	.	.	.

.	.	.	.	.	.	8	0.67	1	1	1	1	4	.	.	.	.	.	.	.	.	.	.	.	.	.	.	.
.	.	.	.	.	.	8	0.67	1	1	1	1	4	.	.	.	.	.	.	.	.	.	.	.	.	.	.	.
.	.	.	.	.	.	10	0.83	1	1	1	0	3	.	.	.	.	.	.	.	.	.	.	.	.	.	.	.
.	.	.	.	.	.	8	0.67	1	1	1	0	3	.	.	.	.	.	.	.	.	.	.	.	.	.	.	.
.	.	.	.	.	.	12	1	1	1	1	1	4	.	.	.	.	.	.	.	.	.	.	.	.	.	.	.
.	.	.	.	.	.	12	1	1	1	1	1	4	.	.	.	.	.	.	.	.	.	.	.	.	.	.	.
.	.	.	.	.	.	12	1	1	1	1	1	4	.	.	.	.	.	.	.	.	.	.	.	.	.	.	.
.	.	.	.	.	.	12	1	1	1	1	1	4	.	.	.	.	.	.	.	.	.	.	.	.	.	.	.
.	.	.	.	.	.	8	0.67	1	1	1	1	4	.	.	.	.	.	.	.	.	.	.	.	.	.	.	.
.	.	.	.	.	.	10	0.83	1	1	1	1	4	.	.	.	.	.	.	.	.	.	.	.	.	.	.	.
.	.	.	.	.	.	10	0.83	0	1	1	1	3	.	.	.	.	.	.	.	.	.	.	.	.	.	.	.
.	.	.	.	.	.	12	1	1	1	1	1	4	.	.	.	.	.	.	.	.	.	.	.	.	.	.	.
.	.	.	.	.	.	9	0.75	1	1	1	1	4	.	.	.	.	.	.	.	.	.	.	.	.	.	.	.
.	.	.	.	.	.	10	0.83	1	1	1	1	4	.	.	.	.	.	.	.	.	.	.	.	.	.	.	.
.	.	.	.	.	.	9	0.75	1	1	0	0	2	.	.	.	.	.	.	.	.	.	.	.	.	.	.	.
.	.	.	.	.	.	8	0.67	1	1	1	1	4	.	.	.	.	.	.	.	.	.	.	.	.	.	.	.
.	.	.	.	.	.	10	0.83	1	1	1	1	4	.	.	.	.	.	.	.	.	.	.	.	.	.	.	.
.	.	.	.	.	.	10	0.83	1	1	0	1	3	.	.	.	.	.	.	.	.	.	.	.	.	.	.	.
.	.	.	.	.	.	12	1	1	1	1	1	4	.	.	.	.	.	.	.	.	.	.	.	.	.	.	.
.	.	.	.	.	.	9	0.75	1	1	1	1	4	.	.	.	.	.	.	.	.	.	.	.	.	.	.	.
.	.	.	.	.	.	8	0.67	1	1	1	1	4	.	.	.	.	.	.	.	.	.	.	.	.	.	.	.
.	.	.	.	.	.	12	1	1	1	1	1	4	.	.	.	.	.	.	.	.	.	.	.	.	.	.	.
.	.	.	.	.	.	10	0.83	1	1	1	1	4	.	.	.	.	.	.	.	.	.	.	.	.	.	.	.
.	.	.	.	.	.	9	0.75	1	1	1	0	3	.	.	.	.	.	.	.	.	.	.	.	.	.	.	.
.	.	.	.	.	.	8	0.67	0	1	0	1	2	.	.	.	.	.	.	.	.	.	.	.	.	.	.	.
.	.	.	.	.	.	10	0.83	0	1	1	1	3	.	.	.	.	.	.	.	.	.	.	.	.	.	.	.
.	.	.	.	.	.	12	1	1	1	1	1	4	.	.	.	.	.	.	.	.	.	.	.	.	.	.	.
.	.	.	.	.	.	12	1	1	1	1	1	4	.	.	.	.	.	.	.	.	.	.	.	.	.	.	.
.	.	.	.	.	.	9	0.75	1	1	1	1	4	.	.	.	.	.	.	.	.	.	.	.	.	.	.	.
.	.	.	.	.	.	12	1	1	1	1	1	4	.	.	.	.	.	.	.	.	.	.	.	.	.	.	.
.	.	.	.	.	.	10	0.83	1	1	1	1	4	.	.	.	.	.	.	.	.	.	.	.	.	.	.	.
.	.	.	.	.	.	9	0.75	1	1	1	1	4	.	.	.	.	.	.	.	.	.	.	.	.	.	.	.
.	.	.	.	.	.	12	1	1	1	1	1	4	.	.	.	.	.	.	.	.	.	.	.	.	.	.	.
.	.	.	.	.	.	8	0.67	0	1	1	1	3	.	.	.	.	.	.	.	.	.	.	.	.	.	.	.
.	.	.	.	.	.	11	0.92	1	1	1	1	4	.	.	.	.	.	.	.	.	.	.	.	.	.	.	.
1	1	0	0	0	0	7	0.58	1	1	1	1	4	1	1	1	1	1	1	1	1	1	1	1	1	1	1	1
1	1	1	0	0	0	7	0.58	1	1	1	1	4	1	0	1	1	1	1	1	1	1	1	0	0	0	0	0
1	1	0	0	1	1	9	0.75	1	1	1	1	4	1	1	1	0	0	1	1	0	1	1	1	1	1	1	1
1	1	0	0	0	0	7	0.58	1	1	1	1	4	1	1	1	1	1	1	1	1	1	1	1	1	1	1	1
0	1	0	0	0	1	6	0.5	1	1	1	1	4	1	0	1	1	0	1	0	1	0	0	0	0	0	0	0
1	1	0	1	0	0	8	0.67	1	1	1	1	4	1	1	1	1	1	1	1	1	1	1	1	1	1	1	1
1	1	1	1	0	1	10	0.83	1	1	1	1	4	1	1	1	1	1	0	1	1	1	1	1	1	1	1	1
1	1	1	1	1	0	10	0.83	1	1	1	1	4	1	1	1	1	1	0	1	1	1	1	1	1	1	1	1
0	0	0	0	0	0	3	0.25	1	1	1	1	4	0	0	0	0	0	1	0	0	0	0	0	0	0	0	0
1	1	0	0	1	0	7	0.58	1	1	1	1	4	0	0	0	0	0	0	0	0	1	0	1	0	0	1	1
1	1	1	0	0	1	9	0.75	1	1	1	1	4	1	1	1	1	1	1	1	1	1	1	1	1	1	1	1
1	1	0	0	0	0	7	0.58	1	1	1	1	4	0	0	0	0	0	0	0	0	0	0	0	0	0	0	0
1	1	1	0	1	1	10	0.83	1	1	1	1	4	1	1	1	1	1	1	1	1	1	1	1	1	1	1	1
1	1	1	1	1	1	11	0.92	1	1	1	1	4	1	1	1	1	1	1	1	1	1	1	1	1	1	1	1
0	0	1	0	0	0	4	0.33	1	1	1	1	4	1	1	0	0	0	0	0	0	0	0	0	0	0	0	0
1	0	1	0	1	1	8	0.67	0	1	1	1	3	1	1	1	1	1	1	1	1	1	1	1	1	0	1	1
1	1	1	1	1	1	11	0.92	1	1	1	1	4	1	1	1	1	1	1	1	1	1	1	1	1	1	1	1
0	1	0	0	1	1	8	0.67	1	1	1	1	4	1	1	1	1	1	0	0	0	0	0	0	0	0	0	0
0	1	0	0	1	1	6	0.5	1	1	0	1	3	1	0	0	1	0	0	1	0	0	1	0	0	0	1	0
1	1	1	1	1	1	11	0.92	1	1	1	1	4	1	1	1	1	1	1	1	1	1	1	1	1	1	1	1
1	1	0	0	0	0	6	0.5	1	1	1	1	4	1	1	1	1	1	1	1	1	1	1	1	1	1	1	1
1	1	0	0	0	0	7	0.58	0	1	1	1	3	1	0	0	0	0	0	0	0	0	0	0	1	0	0	0
1	1	0	0	0	0	7	0.58	1	1	1	1	4	1	0	0	0	0	0	0	0	0	0	0	1	0	0	0
1	1	1	1	1	1	11	0.92	1	0	1	1	3	1	1	1	1	1	1	1	1	1	1	1	1	1	1	1
1	1	0	1	0	1	8	0.67	1	1	1	1	4	1	1	1	1	0	0	1	1	1	1	1	1	0	1	0
0	1	1	1	1	1	7	0.58	1	1	1	1	4	1	1	0	1	1	1	1	1	1	1	1	1	0	1	1
1	1	0	0	0	0	7	0.58	1	1	1	1	4	1	0	1	0	1	1	0	1	1	1	0	1	1	0	1
1	1	1	0	0	0	8	0.67	0	1	1	1	3	1	0	1	1	1	0	1	1	1	1	1	0	0	1	1
1	1	1	0	1	1	9	0.75	1	1	1	1	4	1	0	1	1	1	1	1	1	1	0	0	1	0	0	0
1	0	0	0	0	1	5	0.42	1	1	1	0	3	1	0	1	1	1	1	0	1	1	1	1	1	0	1	1
1	1	0	0	0	0	6	0.5	0	1	1	1	3	1	1	1	1	1	1	1	1	1	1	1	1	1	1	1
1	1	1	0	0	1	9	0.75	1	1	1	1	4	1	1	1	1	1	1	1	1	1	0	1	0	0	1	0
1	1	1	1	0	1	10	0.83	0	1	1	1	3	1	1	1	1	1	0	0	0	0	0	0	0	0	1	0
1	1	1	1	0	1	10	0.83	1	1	1	1	4	1	1	1	1	1	1	1	1	1	1	1	1	0	0	0
1	1	1	1	1	1	11	0.92	1	1	1	1	4	1	1	1	1	1	1	1	1	1	1	1	1	1	1	1

arith_16	arith_17	arith_18	arith_sum	arith_pomp	msr_pomp	ttr_t1	KRT	ttr_t2	KRT.z	TTR_t2.z	cmas	cmas.z
0	0	0	1	0.06	0.32	1	21	10	-0.45	-1.41	-1.86	0.14
1	1	1	15	0.83	0.77	0	44	23	1.48	0.58	2.06	0.85
1	1	1	17	0.94	0.8	6	34	31	0.64	1.8	2.44	0.88
1	0	1	14	0.78	0.78	6	35	32	0.72	1.95	2.68	0.91
1	1	1	16	0.89	0.91	22	48	31	1.82	1.8	3.62	0.97
1	1	1	18	1	0.86	4	16	16	-0.88	-0.49	-1.37	0.25
1	1	1	18	1	0.82	7	28	27	0.14	1.19	1.33	0.77
0	0	0	4	0.22	0.51	2	19	20	-0.62	0.12	-0.5	0.43
0	0	0	0	0	0.55	0	15	11	-0.96	-1.25	-2.21	0.09
0	1	1	17	0.94	0.69	3	18	14	-0.71	-0.79	-1.5	0.22
0	0	0	9	0.5	0.59	4	19	12	-0.62	-1.1	-1.72	0.17
0	1	1	17	0.94	0.74	4	32	21	0.47	0.27	0.75	0.68
1	1	1	18	1	0.79	0	13	18	-1.13	-0.18	-1.31	0.27
1	1	1	18	1	0.83	6	31	20	0.39	0.12	0.51	0.63
0	0	0	2	0.11	0.55	0	21	20	-0.45	0.12	-0.33	0.47
1	1	1	17	0.94	0.84	3	25	18	-0.12	-0.18	-0.3	0.48
0	0	0	9	0.5	0.66	5	35	17	0.72	-0.34	0.39	0.6
0	0	0	2	0.11	0.44	1	23	19	-0.29	-0.03	-0.32	0.48
0	0	0	8	0.44	0.55	0	22	13	-0.37	-0.95	-1.32	0.26
0	1	0	4	0.22	0.49	2	32	23	0.47	0.58	1.05	0.73
0	0	0	11	0.61	0.69	4	15	11	-0.96	-1.25	-2.21	0.09
1	1	1	18	1	0.83	10	46	28	1.65	1.34	2.99	0.94
0	0	1	11	0.61	0.73	4	17	19	-0.79	-0.03	-0.82	0.34
0	0	0	2	0.11	0.56	3	24	20	-0.2	0.12	-0.08	0.53
1	1	1	17	0.94	0.93	14	30	15	0.3	-0.64	-0.34	0.47
1	1	1	18	1	0.89	8	50	23	1.99	0.58	2.57	0.9
1	1	1	18	1	0.88	9	35	19	0.72	-0.03	0.69	0.67
1	1	1	18	1	0.97	13	43	28	1.4	1.34	2.74	0.92
1	1	1	17	0.94	0.93	5	32	20	0.47	0.12	0.59	0.65
1	1	1	17	0.94	0.9	6	26	17	-0.03	-0.34	-0.37	0.45
1	1	1	17	0.94	0.86	6	38	29	0.98	1.5	2.47	0.89
1	1	0	15	0.83	0.86	14	32	23	0.47	0.58	1.05	0.73
1	1	1	18	1	0.97	14	48	20	1.82	0.12	1.94	0.84
0	0	0	17	0.94	0.67	11	23	27	-0.29	1.19	0.9	0.7
0	1	1	10	0.56	0.52	0	27	17	0.05	-0.34	-0.29	0.49
1	1	1	18	1	0.77	0	23	32	-0.29	1.95	1.67	0.81
0	0	0	14	0.78	0.78	9	19	20	-0.62	0.12	-0.5	0.43
0	1	1	15	0.83	0.92	5	34	21	0.64	0.27	0.91	0.7
0	0	1	11	0.61	0.56	6	28	19	0.14	-0.03	0.1	0.56
0	1	0	2	0.11	0.47	8	34	21	0.64	0.27	0.91	0.7
1	1	1	16	0.89	0.88	6	28	14	0.14	-0.79	-0.66	0.39
1	1	1	16	0.89	0.88	14	43	20	1.4	0.12	1.52	0.79
1	1	1	17	0.94	0.95	13	35	17	0.72	-0.34	0.39	0.6
1	1	1	14	0.78	0.81	5	26	12	-0.03	-1.1	-1.13	0.29
1	1	1	18	1	0.74	0	16	13	-0.88	-0.95	-1.82	0.14
0	0	0	6	0.33	0.63	5	26	14	-0.03	-0.79	-0.83	0.34
0	0	0	11	0.61	0.7	1	15	13	-0.96	-0.95	-1.91	0.13
1	1	1	18	1	0.7	0	35	21	0.72	0.27	1	0.72
0	0	0	1	0.06	0.26	0	3	8	-1.97	-1.71	-3.68	0.01
1	0	0	2	0.11	0.36	5	18	9	-0.71	-1.56	-2.27	0.08
0	0	1	3	0.17	0.35	0	5	11	-1.8	-1.25	-3.06	0.03
0	0	0	0	0	0.37	0	3	4	-1.97	-2.32	-4.29	0
1	1	1	18	1	0.76	7	39	16	1.06	-0.49	0.57	0.64
1	1	1	16	0.89	0.82	6	29	11	0.22	-1.25	-1.03	0.31
1	1	1	17	0.94	0.83	7	14	13	-1.04	-0.95	-1.99	0.11
1	1	1	18	1	0.86	10	29	21	0.22	0.27	0.49	0.63
0	0	1	8	0.44	0.6	2	14	16	-1.04	-0.49	-1.53	0.2
0	0	0	5	0.28	0.61	5	10	9	-1.38	-1.56	-2.94	0.04
1	1	1	15	0.83	0.74	6	19	17	-0.62	-0.34	-0.96	0.33
1	1	1	18	1	0.79	5	33	23	0.56	0.58	1.14	0.75
1	1	1	17	0.94	0.95	26	53	32	2.24	1.95	4.19	0.99
0	0	0	2	0.11	0.68	14	42	23	1.31	0.58	1.89	0.84
1	1	1	14	0.78	0.78	3	9	17	-1.47	-0.34	-1.8	0.16
0	1	1	17	0.94	0.9	10	26	22	-0.03	0.43	0.39	0.61
1	1	1	18	1	0.97	17	48	24	1.82	0.73	2.55	0.9
1	1	1	18	1	0.97	15	43	23	1.4	0.58	1.98	0.85
1	1	1	17	0.94	0.78	7	16	16	-0.88	-0.49	-1.37	0.25
1	1	1	15	0.83	0.68	12	28	19	0.14	-0.03	0.1	0.56

0	1	1	13	0.72	0.85	7	22	24	-0.37	0.73	0.36	0.59
1	1	1	18	1	0.97	16	37	17	0.89	-0.34	0.56	0.64
0	0	0	4	0.22	0.49	3	40	21	1.15	0.27	1.42	0.78
0	0	0	12	0.67	0.66	6	28	23	0.14	0.58	0.71	0.67
0	0	0	3	0.17	0.52	4	17	15	-0.79	-0.64	-1.43	0.23
1	1	1	18	1	0.97	18	38	31	0.98	1.8	2.78	0.92
1	1	1	15	0.83	0.89	4	25	26	-0.12	1.04	0.92	0.71
1	1	1	14	0.78	0.81	10	44	23	1.48	0.58	2.06	0.85
1	0	0	16	0.89	0.88	5	31	18	0.39	-0.18	0.2	0.58
1	0	1	13	0.72	0.82	6	37	22	0.89	0.43	1.32	0.76
1	1	1	17	0.94	0.95	14	31	13	0.39	-0.95	-0.56	0.41
0	0	0	1	0.06	0.57	2	24	20	-0.2	0.12	-0.08	0.53
0	1	1	9	0.5	0.69	9	18	12	-0.71	-1.1	-1.81	0.15
0	1	1	13	0.72	0.76	0	26	22	-0.03	0.43	0.39	0.61
0	0	0	2	0.11	0.48	3	22	18	-0.37	-0.18	-0.55	0.41
0	0	0	5	0.28	0.49	6	14	16	-1.04	-0.49	-1.53	0.2
0	0	0	7	0.39	0.65	3	11	15	-1.3	-0.64	-1.94	0.13
1	1	1	18	1	0.83	8	28	22	0.14	0.43	0.56	0.64
1	0	0	2	0.11	0.56	0	34	19	0.64	-0.03	0.61	0.65
0	0	0	3	0.17	0.58	3	34	25	0.64	0.88	1.53	0.8
0	1	0	14	0.78	0.79	5	37	23	0.89	0.58	1.47	0.79
1	1	1	18	1	0.97	7	42	13	1.31	-0.95	0.37	0.6
0	0	0	1	0.06	0.59	2	18	11	-0.71	-1.25	-1.96	0.12
1	1	1	16	0.89	0.71	5	27	24	0.05	0.73	0.78	0.68
0	0	0	14	0.78	0.75	5	40	26	1.15	1.04	2.18	0.87
0	0	0	2	0.11	0.56	0	31	24	0.39	0.73	1.12	0.74
0	0	0	4	0.22	0.6	5	14	21	-1.04	0.27	-0.77	0.36
0	0	0	2	0.11	0.53	3	3	10	-1.97	-1.41	-3.38	0.02
0	1	1	17	0.94	0.95	9	49	30	1.9	1.65	3.55	0.97
0	1	1	15	0.83	0.74	9	33	19	0.56	-0.03	0.52	0.63
1	1	1	18	1	0.92	6	39	22	1.06	0.43	1.49	0.79
1	1	1	17	0.94	0.95	28	48	37	1.82	2.72	4.54	0.99
0	1	1	16	0.89	0.94	14	52	38	2.16	2.87	5.03	1
1	1	0	17	0.94	0.93	16	44	20	1.48	0.12	1.6	0.81
1	1	1	18	1	0.91	7	17	20	-0.79	0.12	-0.67	0.38
1	0	1	15	0.83	0.74	7	19	19	-0.62	-0.03	-0.65	0.39
0	0	0	11	0.61	0.84	14	16	20	-0.88	0.12	-0.75	0.36
1	0	0	15	0.83	0.82	12	28	20	0.14	0.12	0.26	0.58
1	1	1	18	1	0.94	12	40	24	1.15	0.73	1.88	0.84
0	0	0	1	0.06	0.51	4	17	18	-0.79	-0.18	-0.98	0.32
0	0	0	6	0.33	0.46	4	16	14	-0.88	-0.79	-1.67	0.18
0	0	0	2	0.11	0.56	2	20	10	-0.54	-1.41	-1.94	0.13
0	0	0	2	0.11	0.56	4	20	16	-0.54	-0.49	-1.03	0.32
0	1	1	17	0.94	0.74	4	22	20	-0.37	0.12	-0.25	0.5
1	1	1	15	0.83	0.86	12	15	26	-0.96	1.04	0.08	0.56
0	1	1	17	0.94	0.83	2	17	15	-0.79	-0.64	-1.43	0.23
1	1	1	18	1	0.85	7	26	16	-0.03	-0.49	-0.52	0.42
0	0	0	13	0.72	0.81	7	23	12	-0.29	-1.1	-1.39	0.24
0	0	0	1	0.06	0.36	1	9	12	-1.47	-1.1	-2.57	0.06
1	1	1	18	1	0.83	10	28	23	0.14	0.58	0.71	0.67
0	0	0	5	0.28	0.61	6	25	18	-0.12	-0.18	-0.3	0.48
0	0	0	1	0.06	0.59	10	26	18	-0.03	-0.18	-0.22	0.51
1	1	1	18	1	0.83	13	27	28	0.05	1.34	1.39	0.78
0	0	0	0	0	0.46	2	11	16	-1.3	-0.49	-1.79	0.16
0	0	0	10	0.56	0.64	2	23	19	-0.29	-0.03	-0.32	0.48
.	.	.	11	0.61	0.75	8	24	20	-0.2	0.12	-0.08	0.53
.	.	.	10	0.56	0.8	13	33	22	0.56	0.43	0.98	0.72
.	.	.	18	1	0.6	7	9	16	-1.47	-0.49	-1.95	0.12
.	.	.	3	0.17	0.49	2	11	28	-1.3	1.34	0.05	0.55
.	.	.	17	0.94	0.56	3	7	20	-1.63	0.12	-1.51	0.21
.	.	.	18	1	0.94	13	30	24	0.3	0.73	1.04	0.73
.	.	.	13	0.72	0.45	0	22	11	-0.37	-1.25	-1.62	0.18
.	.	.	5	0.28	0.64	1	13	12	-1.13	-1.1	-2.23	0.09
.	.	.	5	0.28	0.43	4	31	10	0.39	-1.41	-1.02	0.32
.	.	.	10	0.56	0.71	0	10	7	-1.38	-1.86	-3.24	0.02
.	.	.	14	0.78	0.75	2	40	19	1.15	-0.03	1.11	0.74
.	.	.	18	1	0.91	5	26	17	-0.03	-0.34	-0.37	0.45
.	.	.	6	0.33	0.55	0	10	15	-1.38	-0.64	-2.02	0.11
.	.	.	15	0.83	0.79	4	25	21	-0.12	0.27	0.16	0.57

.	.	.	11	0.61	0.78	10	38	17	0.98	-0.34	0.64	0.66
.	.	.	7	0.39	0.59	6	23	14	-0.29	-0.79	-1.08	0.3
.	.	.	17	0.94	0.8	2	21	21	-0.45	0.27	-0.18	0.52
.	.	.	16	0.89	0.9	2	28	17	0.14	-0.34	-0.2	0.51
.	.	.	0	0	0.27	3	27	17	0.05	-0.34	-0.29	0.49
.	.	.	3	0.17	0.41	0	9	16	-1.47	-0.49	-1.95	0.12
.	.	.	11	0.61	0.75	3	24	17	-0.2	-0.34	-0.54	0.42
.	.	.	2	0.11	0.29	6	7	10	-1.63	-1.41	-3.04	0.03
.	.	.	18	1	0.79	4	39	21	1.06	0.27	1.34	0.77
.	.	.	11	0.61	0.66	5	19	14	-0.62	-0.79	-1.42	0.24
.	.	.	17	0.94	0.9	13	44	18	1.48	-0.18	1.3	0.76
.	.	.	9	0.5	0.62	4	22	14	-0.37	-0.79	-1.17	0.29
.	.	.	18	1	0.74	8	20	15	-0.54	-0.64	-1.18	0.28
.	.	.	18	1	0.97	11	43	21	1.4	0.27	1.67	0.81
.	.	.	0	0	0.52	6	24	11	-0.2	-1.25	-1.45	0.23
.	.	.	8	0.44	0.73	3	22	11	-0.37	-1.25	-1.62	0.18
.	.	.	16	0.89	0.84	5	23	20	-0.29	0.12	-0.16	0.52
.	.	.	7	0.39	0.7	3	19	15	-0.62	-0.64	-1.27	0.27
.	.	.	0	0	0.55	4	12	7	-1.21	-1.86	-3.08	0.03
.	.	.	0	0	0.48	0	12	9	-1.21	-1.56	-2.77	0.04
.	.	.	11	0.61	0.69	1	11	11	-1.3	-1.25	-2.55	0.06
.	.	.	15	0.83	0.73	5	22	12	-0.37	-1.1	-1.47	0.22
.	.	.	2	0.11	0.47	8	18	17	-0.71	-0.34	-1.04	0.31
.	.	.	9	0.5	0.74	0	22	23	-0.37	0.58	0.21	0.58
.	.	.	0	0	0.03	2	22	17	-0.37	-0.34	-0.71	0.37
.	.	.	17	0.94	0.86	4	18	16	-0.71	-0.49	-1.2	0.28
.	.	.	2	0.11	0.36	2	4	2	-1.89	-2.63	-4.51	0
.	.	.	0	0	0.2	0	5	7	-1.8	-1.86	-3.67	0.01
.	.	.	16	0.89	0.88	4	27	15	0.05	-0.64	-0.59	0.4
.	.	.	17	0.94	0.84	10	23	16	-0.29	-0.49	-0.78	0.35
.	.	.	1	0.06	0.6	0	11	14	-1.3	-0.79	-2.09	0.1
.	.	.	6	0.33	0.67	5	16	12	-0.88	-1.1	-1.98	0.12
.	.	.	9	0.5	0.78	7	15	22	-0.96	0.43	-0.53	0.42
.	.	.	1	0.06	0.31	0	23	10	-0.29	-1.41	-1.69	0.17
.	.	.	0	0	0.49	4	16	15	-0.88	-0.64	-1.52	0.21
.	.	.	18	1	0.86	9	32	19	0.47	-0.03	0.44	0.62
.	.	.	0	0	0.39	2	16	13	-0.88	-0.95	-1.82	0.14
.	.	.	18	1	0.89	10	29	20	0.22	0.12	0.34	0.59
.	.	.	18	1	1	9	26	16	-0.03	-0.49	-0.52	0.42
.	.	.	17	0.94	0.98	2	20	15	-0.54	-0.64	-1.18	0.28
.	.	.	5	0.28	0.7	5	26	16	-0.03	-0.49	-0.52	0.42
.	.	.	1	0.06	0.53	2	21	16	-0.45	-0.49	-0.94	0.33
.	.	.	18	1	0.89	5	27	17	0.05	-0.34	-0.29	0.49
.	.	.	12	0.67	0.71	3	21	15	-0.45	-0.64	-1.1	0.3
.	.	.	0	0	0.58	4	18	13	-0.71	-0.95	-1.65	0.18
.	.	.	18	1	0.89	10	20	21	-0.54	0.27	-0.26	0.5
.	.	.	5	0.28	0.68	0	14	16	-1.04	-0.49	-1.53	0.2
.	.	.	0	0	0.29	2	4	6	-1.89	-2.02	-3.9	0.01
.	.	.	12	0.67	0.81	4	17	18	-0.79	-0.18	-0.98	0.32
.	.	.	16	0.89	0.93	7	20	15	-0.54	-0.64	-1.18	0.28
.	.	.	11	0.61	0.69	2	17	11	-0.79	-1.25	-2.04	0.1
.	.	.	16	0.89	0.78	1	18	21	-0.71	0.27	-0.43	0.44
.	.	.	18	1	0.85	6	12	17	-1.21	-0.34	-1.55	0.19
.	.	.	9	0.5	0.74	2	13	17	-1.13	-0.34	-1.47	0.22
.	.	.	12	0.67	0.65	2	14	11	-1.04	-1.25	-2.3	0.08
.	.	.	0	0	0.07	1	10	9	-1.38	-1.56	-2.94	0.04
.	.	.	17	0.94	0.77	3	22	10	-0.37	-1.41	-1.78	0.16
.	.	.	0	0	0.55	7	8	13	-1.55	-0.95	-2.5	0.07
.	.	.	5	0.28	0.64	1	5	13	-1.8	-0.95	-2.75	0.05
.	.	.	16	0.89	0.85	3	29	26	0.22	1.04	1.26	0.75
.	.	.	16	0.89	0.81	6	29	22	0.22	0.43	0.65	0.66
.	.	.	16	0.89	0.88	4	15	16	-0.96	-0.49	-1.45	0.23
.	.	.	18	1	1	12	43	35	1.4	2.41	3.81	0.98
.	.	.	18	1	0.92	8	38	29	0.98	1.5	2.47	0.89
.	.	.	18	1	1	11	49	34	1.9	2.26	4.16	0.98
.	.	.	18	1	0.91	4	23	20	-0.29	0.12	-0.16	0.52
.	.	.	18	1	0.94	19	47	29	1.74	1.5	3.23	0.95
.	.	.	12	0.67	0.79	6	27	26	0.05	1.04	1.09	0.74
.	.	.	18	1	0.82	12	18	24	-0.71	0.73	0.03	0.55
.	.	.	18	1	0.86	6	30	24	0.3	0.73	1.04	0.73

.	.	.	15	0.83	0.71	4	30	18	0.3	-0.18	0.12	0.57
.	.	.	17	0.94	0.81	5	8	14	-1.55	-0.79	-2.34	0.08
.	.	.	17	0.94	0.98	8	52	28	2.16	1.34	3.5	0.96
.	.	.	9	0.5	0.68	0	16	20	-0.88	0.12	-0.75	0.36
.	.	.	18	1	0.8	6	40	32	1.15	1.95	3.1	0.95
.	.	.	16	0.89	0.88	1	33	27	0.56	1.19	1.75	0.82
.	.	.	18	1	0.82	8	24	26	-0.2	1.04	0.84	0.69
.	.	.	17	0.94	0.8	1	25	20	-0.12	0.12	0	0.55
.	.	.	13	0.72	0.8	6	25	26	-0.12	1.04	0.92	0.71
.	.	.	18	1	0.92	7	24	14	-0.2	-0.79	-1	0.32
.	.	.	18	1	0.94	10	46	19	1.65	-0.03	1.62	0.81
.	.	.	14	0.78	0.93	10	39	24	1.06	0.73	1.79	0.83
.	.	.	18	1	0.88	5	27	30	0.05	1.65	1.7	0.82
.	.	.	18	1	0.92	11	29	28	0.22	1.34	1.56	0.8
.	.	.	18	1	0.91	2	34	31	0.64	1.8	2.44	0.88
.	.	.	16	0.89	0.96	15	34	27	0.64	1.19	1.83	0.83
.	.	.	18	1	0.94	7	37	30	0.89	1.65	2.54	0.9
.	.	.	18	1	1	12	39	21	1.06	0.27	1.34	0.77
.	.	.	18	1	1	10	33	26	0.56	1.04	1.59	0.8
.	.	.	11	0.61	0.81	2	14	17	-1.04	-0.34	-1.38	0.25
.	.	.	14	0.78	0.84	6	27	18	0.05	-0.18	-0.13	0.52
.	.	.	11	0.61	0.78	7	14	15	-1.04	-0.64	-1.69	0.17
.	.	.	18	1	0.89	6	23	16	-0.29	-0.49	-0.78	0.35
.	.	.	18	1	0.89	11	31	33	0.39	2.11	2.49	0.89
.	.	.	12	0.67	0.71	3	11	19	-1.3	-0.03	-1.33	0.26
.	.	.	18	1	1	21	45	32	1.57	1.95	3.52	0.97
.	.	.	18	1	1	15	36	18	0.81	-0.18	0.62	0.66
.	.	.	18	1	0.91	2	23	19	-0.29	-0.03	-0.32	0.48
.	.	.	18	1	0.91	7	16	15	-0.88	-0.64	-1.52	0.21
.	.	.	18	1	0.91	7	45	25	1.57	0.88	2.45	0.88
.	.	.	6	0.33	0.57	5	23	8	-0.29	-1.71	-2	0.11
.	.	.	18	1	0.79	2	20	19	-0.54	-0.03	-0.57	0.41
.	.	.	18	1	0.81	12	22	28	-0.37	1.34	0.97	0.72
.	.	.	18	1	0.84	3	11	19	-1.3	-0.03	-1.33	0.26
.	.	.	15	0.83	0.77	1	25	15	-0.12	-0.64	-0.76	0.36
.	.	.	16	0.89	0.9	5	26	15	-0.03	-0.64	-0.68	0.37
.	.	.	3	0.17	0.49	1	5	13	-1.8	-0.95	-2.75	0.05
.	.	.	5	0.28	0.59	1	18	15	-0.71	-0.64	-1.35	0.26
.	.	.	2	0.11	0.46	0	7	10	-1.63	-1.41	-3.04	0.03
.	.	.	16	0.89	0.66	3	6	15	-1.72	-0.64	-2.36	0.07
.	.	.	17	0.94	0.8	2	22	24	-0.37	0.73	0.36	0.59
.	.	.	11	0.61	0.65	6	34	21	0.64	0.27	0.91	0.7
.	.	.	18	1	0.83	1	16	8	-0.88	-1.71	-2.59	0.06
.	.	.	17	0.94	0.83	7	23	23	-0.29	0.58	0.29	0.59
.	.	.	10	0.56	0.7	2	19	21	-0.62	0.27	-0.35	0.46
.	.	.	15	0.83	0.86	2	16	15	-0.88	-0.64	-1.52	0.21
.	.	.	18	1	0.79	7	19	21	-0.62	0.27	-0.35	0.46
.	.	.	17	0.94	0.93	7	26	19	-0.03	-0.03	-0.06	0.54
.	.	.	17	0.94	0.95	4	16	13	-0.88	-0.95	-1.82	0.14
.	.	.	17	0.94	0.8	7	28	19	0.14	-0.03	0.1	0.56
.	.	.	11	0.61	0.66	3	21	15	-0.45	-0.64	-1.1	0.3
.	.	.	17	0.94	0.9	11	17	20	-0.79	0.12	-0.67	0.38
.	.	.	12	0.67	0.71	1	15	14	-0.96	-0.79	-1.75	0.17
.	.	.	16	0.89	0.85	8	17	20	-0.79	0.12	-0.67	0.38
.	.	.	17	0.94	0.89	16	46	20	1.65	0.12	1.77	0.83
.	.	.	18	1	0.82	7	24	19	-0.2	-0.03	-0.23	0.5
.	.	.	18	1	0.82	4	13	21	-1.13	0.27	-0.85	0.34
.	.	.	17	0.94	0.71	3	12	10	-1.21	-1.41	-2.62	0.05
.	.	.	17	0.94	0.76	11	22	19	-0.37	-0.03	-0.4	0.45
.	.	.	18	1	0.79	10	26	15	-0.03	-0.64	-0.68	0.37
.	.	.	17	0.94	0.86	4	13	12	-1.13	-1.1	-2.23	0.09
.	.	.	18	1	0.94	10	26	29	-0.03	1.5	1.46	0.79
.	.	.	12	0.67	0.89	1	15	11	-0.96	-1.25	-2.21	0.09
.	.	.	11	0.61	0.78	11	5	14	-1.8	-0.79	-2.6	0.06
.	.	.	18	1	1	15	44	35	1.48	2.41	3.89	0.98
.	.	.	11	0.61	0.75	2	10	5	-1.38	-2.17	-3.55	0.01
.	.	.	18	1	0.88	0	22	16	-0.37	-0.49	-0.86	0.34
.	.	.	18	1	0.82	6	19	18	-0.62	-0.18	-0.81	0.35
.	.	.	16	0.89	0.9	4	17	22	-0.79	0.43	-0.36	0.46

.	.	.	5	0.28	0.48	0	11	7	-1.3	-1.86	-3.16	0.02
.	.	.	17	0.94	0.87	5	35	15	0.72	-0.64	0.08	0.56
.	.	.	9	0.5	0.78	10	25	16	-0.12	-0.49	-0.61	0.4
.	.	.	3	0.17	0.54	2	26	12	-0.03	-1.1	-1.13	0.29
.	.	.	18	1	1	14	54	26	2.33	1.04	3.36	0.96
.	.	.	18	1	1	16	52	24	2.16	0.73	2.89	0.93
.	.	.	18	1	1	18	49	36	1.9	2.56	4.47	0.99
.	.	.	2	0.11	0.7	15	51	23	2.07	0.58	2.65	0.91
.	.	.	4	0.22	0.6	6	36	19	0.81	-0.03	0.78	0.68
.	.	.	1	0.06	0.63	7	37	13	0.89	-0.95	-0.05	0.54
.	.	.	18	1	0.94	11	48	22	1.82	0.43	2.25	0.87
.	.	.	4	0.22	0.71	2	12	15	-1.21	-0.64	-1.85	0.14
.	.	.	1	0.06	0.57	0	45	15	1.57	-0.64	0.92	0.72
.	.	.	3	0.17	0.63	5	44	23	1.48	0.58	2.06	0.85
.	.	.	5	0.28	0.68	0	44	13	1.48	-0.95	0.54	0.63
.	.	.	2	0.11	0.59	4	12	15	-1.21	-0.64	-1.85	0.14
.	.	.	2	0.11	0.61	4	44	23	1.48	0.58	2.06	0.85
.	.	.	1	0.06	0.56	0	33	6	0.56	-2.02	-1.46	0.23
.	.	.	13	0.72	0.91	5	41	17	1.23	-0.34	0.89	0.7
.	.	.	5	0.28	0.61	2	19	13	-0.62	-0.95	-1.57	0.19
.	.	.	1	0.06	0.54	3	29	14	0.22	-0.79	-0.58	0.4
.	.	.	9	0.5	0.77	8	29	14	0.22	-0.79	-0.58	0.4
.	.	.	11	0.61	0.75	11	42	29	1.31	1.5	2.81	0.92
.	.	.	0	0	0.48	2	39	16	1.06	-0.49	0.57	0.64
.	.	.	0	0	0.42	5	11	11	-1.3	-1.25	-2.55	0.06
.	.	.	10	0.56	0.73	3	38	24	0.98	0.73	1.71	0.82
.	.	.	18	1	1	13	40	26	1.15	1.04	2.18	0.87
.	.	.	7	0.39	0.8	13	41	27	1.23	1.19	2.42	0.88
.	.	.	4	0.22	0.59	8	34	27	0.64	1.19	1.83	0.83
.	.	.	18	1	1	10	47	26	1.74	1.04	2.77	0.92
.	.	.	18	1	0.91	15	47	29	1.74	1.5	3.23	0.95
.	.	.	18	1	0.88	7	29	15	0.22	-0.64	-0.42	0.45
.	.	.	18	1	1	16	47	28	1.74	1.34	3.08	0.94
.	.	.	1	0.06	0.47	1	8	14	-1.55	-0.79	-2.34	0.08
.	.	.	18	1	0.97	12	38	16	0.98	-0.49	0.49	0.62
1	0	1	17	0.94	0.78	5	43	29	1.4	1.5	2.89	0.93
0	0	0	9	0.5	0.63	4	17	20	-0.79	0.12	-0.67	0.38
1	1	1	15	0.83	0.79	9	18	17	-0.71	-0.34	-1.04	0.31
0	1	1	17	0.94	0.78	13	34	23	0.64	0.58	1.22	0.75
0	0	0	5	0.28	0.49	11	25	17	-0.12	-0.34	-0.45	0.44
1	0	0	16	0.89	0.82	7	32	30	0.47	1.65	2.12	0.86
1	0	1	18	1	0.84	8	33	24	0.56	0.73	1.29	0.76
1	0	1	16	0.89	0.91	15	21	20	-0.45	0.12	-0.33	0.47
0	0	0	0	0	0.32	3	28	23	0.14	0.58	0.71	0.67
1	0	0	4	0.22	0.57	8	33	15	0.56	-0.64	-0.09	0.53
1	1	1	18	1	0.88	15	45	29	1.57	1.5	3.06	0.94
0	0	0	0	0	0.49	14	35	31	0.72	1.8	2.53	0.9
1	1	0	17	0.94	0.93	11	28	27	0.14	1.19	1.33	0.77
1	1	1	18	1	0.97	14	51	37	2.07	2.72	4.79	1
0	0	0	2	0.11	0.41	1	15	18	-0.96	-0.18	-1.14	0.29
0	0	1	15	0.83	0.83	6	28	14	0.14	-0.79	-0.66	0.39
1	1	1	18	1	0.97	23	56	34	2.49	2.26	4.75	0.99
0	0	0	5	0.28	0.61	2	28	17	0.14	-0.34	-0.2	0.51
0	0	1	6	0.33	0.51	1	15	17	-0.96	-0.34	-1.3	0.27
1	1	1	18	1	0.97	11	40	31	1.15	1.8	2.95	0.94
1	1	1	18	1	0.77	12	27	22	0.05	0.43	0.48	0.62
0	0	0	1	0.06	0.55	4	30	14	0.3	-0.79	-0.49	0.44
0	0	0	2	0.11	0.5	0	19	13	-0.62	-0.95	-1.57	0.19
1	1	1	18	1	0.97	14	49	30	1.9	1.65	3.55	0.97
0	1	1	13	0.72	0.7	0	28	21	0.14	0.27	0.41	0.61
1	1	1	16	0.89	0.76	0	25	29	-0.12	1.5	1.38	0.78
1	1	1	13	0.72	0.74	6	25	24	-0.12	0.73	0.61	0.66
0	1	1	13	0.72	0.73	7	30	17	0.3	-0.34	-0.03	0.54
0	1	1	11	0.61	0.69	5	26	25	-0.03	0.88	0.85	0.69
1	1	1	16	0.89	0.74	6	26	22	-0.03	0.43	0.39	0.61
1	1	0	17	0.94	0.78	11	32	29	0.47	1.5	1.97	0.85
0	1	1	12	0.67	0.74	4	47	25	1.74	0.88	2.62	0.91
0	0	0	5	0.28	0.7	12	45	31	1.57	1.8	3.37	0.96
0	0	0	12	0.67	0.83	6	28	24	0.14	0.73	0.87	0.69
1	1	1	18	1	0.97	16	47	27	1.74	1.19	2.93	0.93
