# Supplementary material for: Assessing Mathematical School Readiness
Source: Front Psychol. 2019 May 24;10:1173. doi: 10.3389/fpsyg.2019.01173 (PMC6543806; doi:10.3389/fpsyg.2019.01173)
Supplement: APPENDIX A — Inital version of the “mathematical school readiness test”; only 3 items are included in the final version of the test. [file Data_Sheet_2.docx]

Appendix A

CAUTION : DO NOT USE THIS TASK

**
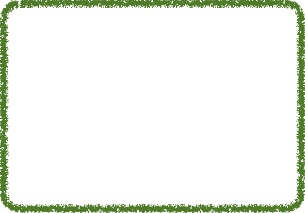

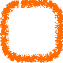

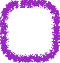

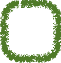

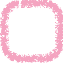

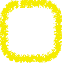

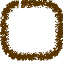

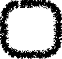

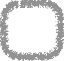

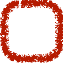
**

Entoure les chiffres et barre si ce n’est pas un chiffre.

3 6

f 8

9

a @

$

..... / 8

Ecris le chiffre dans la bonne case!

Dans le bleu, écrivez 4 (...)


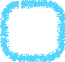


..... / 10


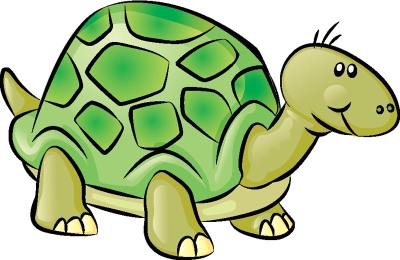

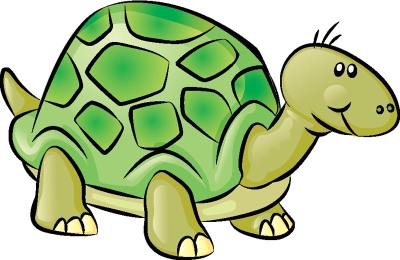

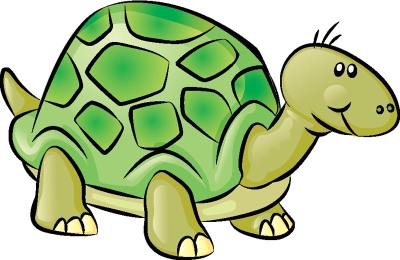

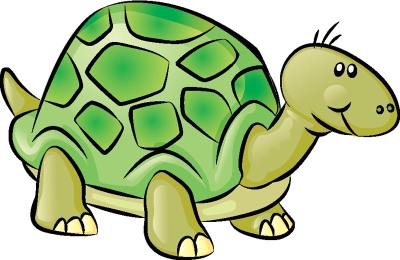

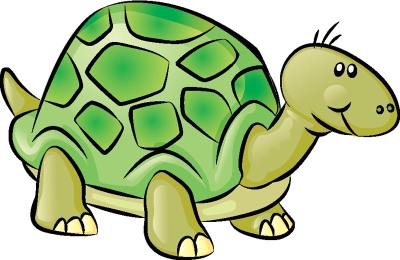

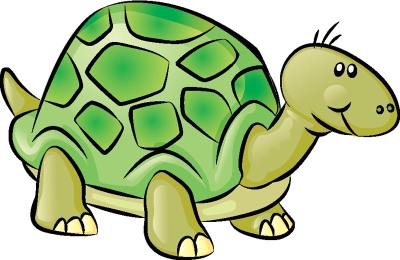

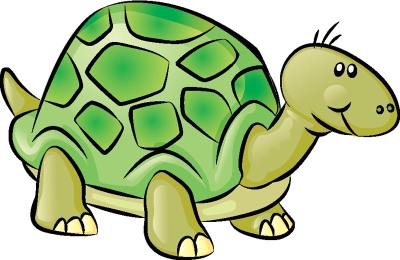

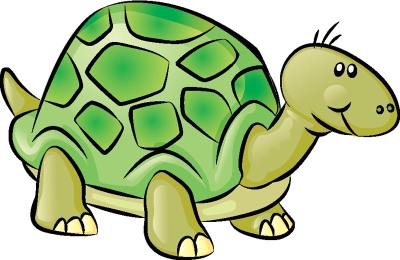

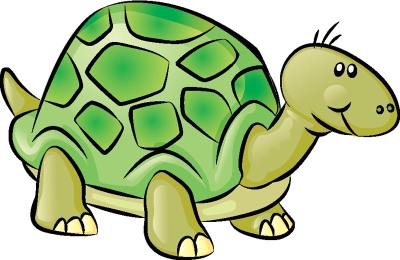

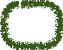


CAUTION : DO NOT USE THIS TASK

Entoure le plus grand nombre! :

2 6


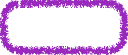


4 5


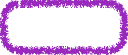


8 7


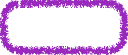


9 3


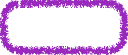


16 11


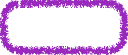


13 14


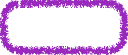


60 50


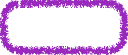


40 90


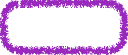


59 73


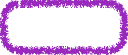


42 38


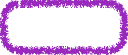


109 180


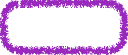


403 420


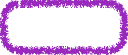


Combien y a-t-il de tortues?

..... / 12

..... / 4


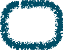

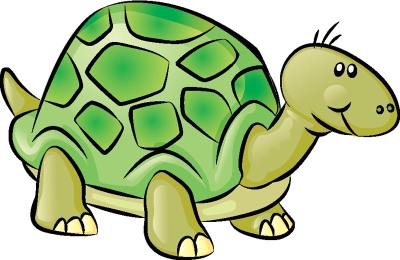

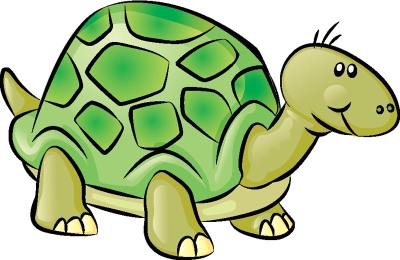

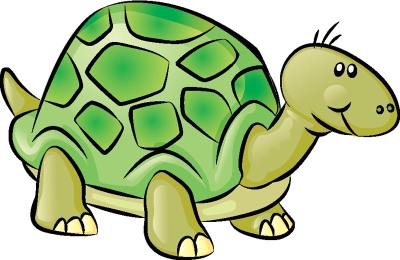

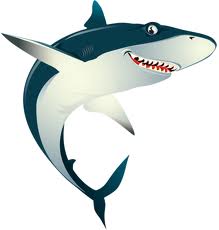

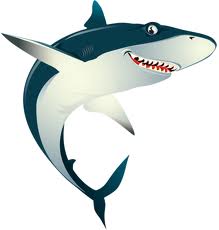

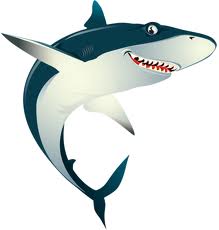

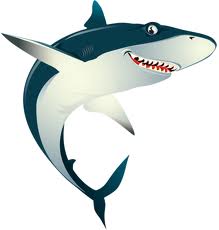

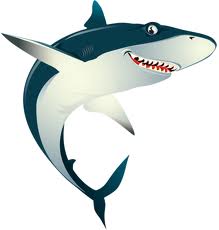

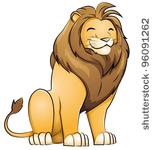

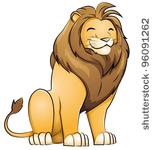

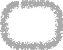

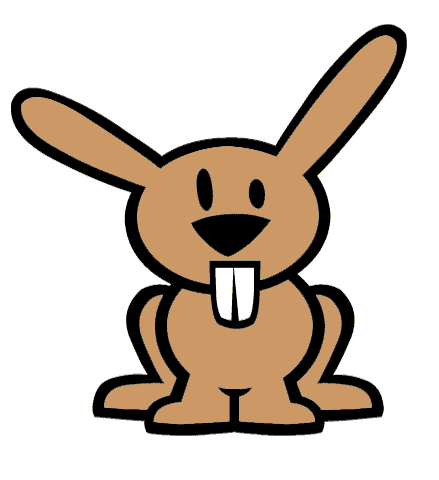

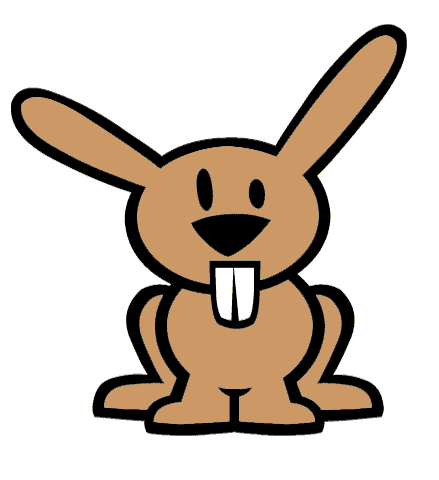

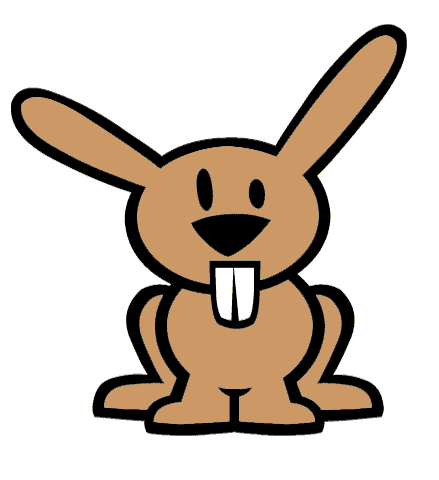

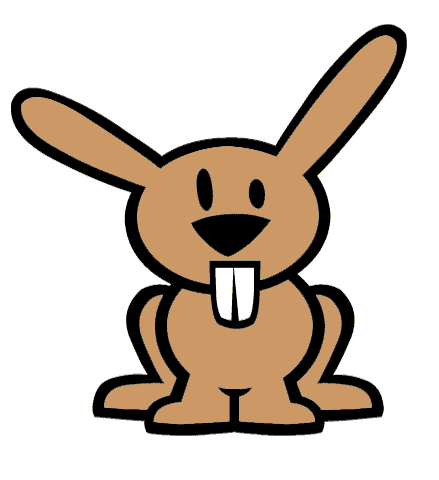

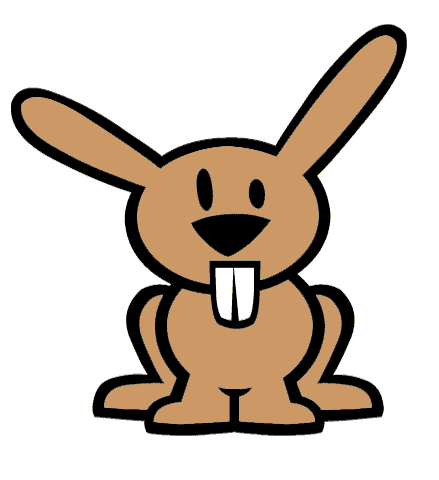

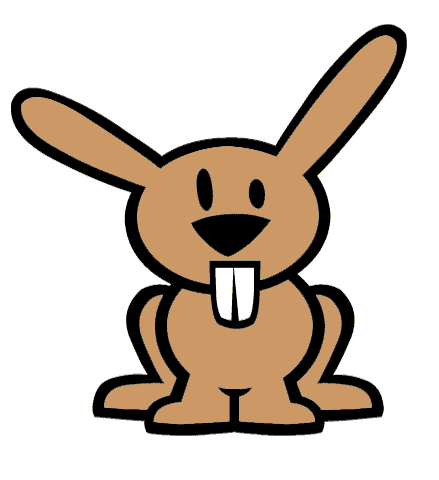

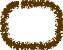

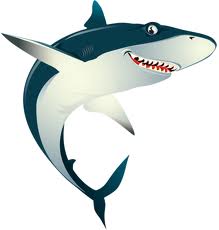


CAUTION : DO NOT USE THIS TASK

CAUTION : DO NOT USE THIS TASK

Combien y a-t-il d’animaux en tout?

Combien y a-t-il de requins ?

Combien y a-t-il de lapins?


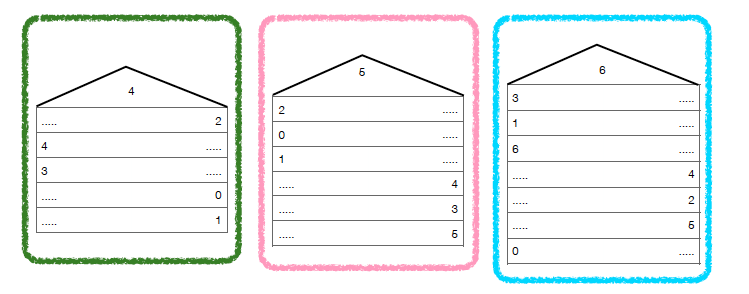


..... / 5

La maison de 4


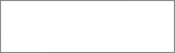


La maison de 5


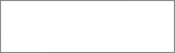


La maison de 6


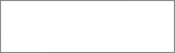


Arithmétique


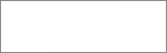


..... / 6

..... / 7


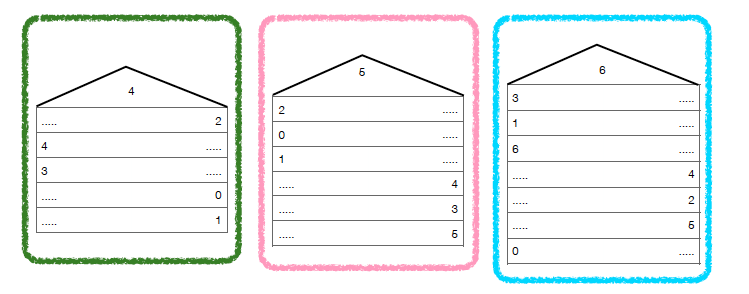


..... / 5

La maison de 4


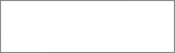


La maison de 5


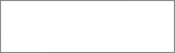


La maison de 6


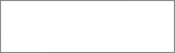


Arithmétique


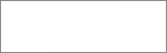


..... / 6

..... / 7
